# Antibacterial Activity of Small Molecules Which Eradicate Methicillin-Resistant *Staphylococcus aureus* Persisters

**DOI:** 10.3389/fmicb.2022.823394

**Published:** 2022-02-01

**Authors:** Mohamad Hamad, Farah Al-Marzooq, Vunnam Srinivasulu, Hany A. Omar, Ashna Sulaiman, Dana M. Zaher, Gorka Orive, Taleb H. Al-Tel

**Affiliations:** ^1^Sharjah Institute for Medical Research, University of Sharjah, Sharjah, United Arab Emirates; ^2^College of Health Sciences, University of Sharjah, Sharjah, United Arab Emirates; ^3^College of Medicine and Health Sciences, UAE University, Al Ain, United Arab Emirates; ^4^College of Pharmacy, University of Sharjah, Sharjah, United Arab Emirates; ^5^NanoBioCel Group, Laboratory of Pharmaceutics, School of Pharmacy, University of the Basque Country (UPV/EHU), Bilbao, Spain

**Keywords:** bacterial resistance, antibacterial, MRSA, persisters, multi-drug resistance, *Staphylococcus aureus*

## Abstract

The serious challenge posed by multidrug-resistant bacterial infections with concomitant treatment failure and high mortality rates presents an urgent threat to the global health. We herein report the discovery of a new class of potent antimicrobial compounds that are highly effective against Gram-positive bacteria, including methicillin-resistant *Staphylococcus aureus* (MRSA). The compounds were efficiently synthesized in one-pot employing a cascade of Groebke–Blackburn–Bienaymé and aza-Michael addition reactions. Phenotypic screening of the pilot library against various bacterial species including methicillin-sensitive and MRSA strains, has identified potent chemotypes with minimal inhibitory concentrations (MIC) of 3.125–6.25 μg/ml. The most potent compounds were fast-acting at eradicating exponentially growing MRSA, with killing achieved after 30 min of exposure to the compounds. They were also able to kill MRSA persister cells which are tolerant to most available medications. Microscopic analysis using fluorescence microscope and atomic force microscope indicate that these compounds lead to disruption of bacterial cell envelopes. Most notably, bacterial resistance toward these compounds was not observed after 20 serial passages in stark contrast to the significant resistance developed rapidly upon exposure to a clinically relevant antibiotic. Furthermore, the compounds did not induce significant hemolysis to human red blood cells. *In vivo* safety studies revealed a high safety profile of these motifs. These small molecules hold a promise for further studies and development as new antibacterial agents against MRSA infections.

## Graphical Abstract

Antibacterial activity of small molecules which eradicate methicillin-resistant *Staphylococcus aureus* (MRSA) persisters.

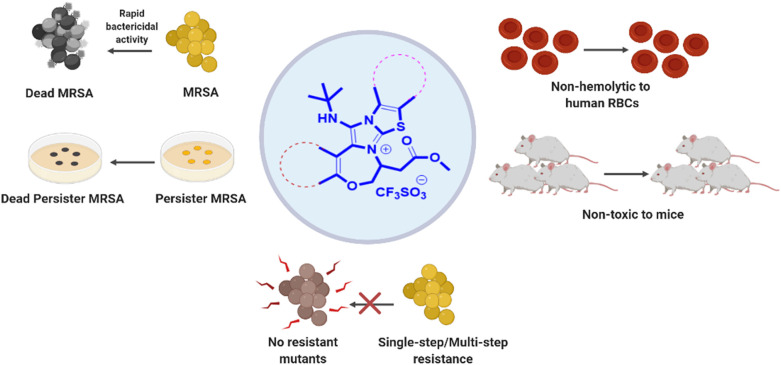

## Introduction

The ongoing COVID-19 pandemic is a wake-up call to seriously reconsider our preparedness to tackle infections (Aghila Rani et al., 2021). The groundbreaking discovery of antibiotics has revolutionized modern medicine and saved countless human lives. Yet, our current arsenal of effective antibiotics is rapidly depleting due to the spread of the multidrug-resistant (MDR) bacteria (Bragginton and Piddock, 2014; Hamad et al., 2019). One of the greatest global public health concerns is the return to the pre-antibiotic era, whereby common infections, minor injuries, or routine surgical procedures, which have been manageable for decades, can once again become fatal (Gupta et al., 2018; Hamad et al., 2019).

The World Health Organization (WHO) has declared a list of priority MDR pathogens causing high morbidity and mortality worldwide as priority targets for the discovery and development of novel antimicrobial drugs (WHO, 2017; Tacconelli et al., 2018). One of the priority MDR pathogens is methicillin-resistant *Staphylococcus aureus* (MRSA), a Gram-positive bacterial pathogen that causes community and healthcare-associated infections with higher rates of morbidity and mortality in comparison to methicillin-sensitive *S. aureus* (Gardete and Tomasz, 2014; Turner et al., 2019). Current antibiotics used for MRSA treatment are vancomycin, daptomycin, and linezolid; however, resistance has emerged against all three antibiotics (Gu et al., 2013; Gardete and Tomasz, 2014; Roch et al., 2017). In addition to its resistance to antibiotics, treatment of MRSA infections is further complicated by its ability to form drug tolerant biofilms and persister cells (Lewis, 2005). Drug-tolerant persisters have low metabolic and biosynthetic rates, which are usually the targets for most antibiotics (Lewis, 2010). The low energy state of persisters also prevents energy-dependent uptake of some antimicrobials such as aminoglycosides (Allison et al., 2011). The frequency of persister cell formation is high during stationary-phase, stress conditions, and biofilms states. Since bacteria often encounter these conditions during host infection, persisters are clinically relevant as contributors to chronic and relapsing infections as well as the development of antibiotic resistance (Fauvart et al., 2011). More recent evidence demonstrates that *Staphylococcus aureus* can also form persisters intracellularly upon antibiotic exposure (Peyrusson et al., 2020).

Currently available antibiotics are based around a limited number of structural classes which inhibit the function of a small number of bacterial targets (Hutchings et al., 2019). Although new derivatives of an existing class can improve treatment, underlying resistance mechanisms against a certain class can sometimes develop to work against newer derivatives (Coates et al., 2011; Cherny et al., 2021). Therefore, there is an urgent need for the discovery of new antibacterial agents with a new mechanism of action that interfere with novel targets and can treat both antibiotic resistant and tolerant MRSA bacteria (Gupta et al., 2018; Kim et al., 2018a).

We have recently reported the construction of scaffolds (Al Tel et al., 2020; Srinivasulu et al., 2021) inspired by the multifaceted biological significance of benzoxazepines (Jaiprakash et al., 2015) and imidazopyridine analogs (Bagdi et al., 2015; Fascio et al., 2015). Such structural options display a broad spectrum of significant biological activities, including a range of central nervous system disorders, anti-inflammatory, anti-Alzheimer, anticancer, antimicrobial, and antiviral activities (Feng et al., 2015; Fox et al., 2015; Jaiprakash et al., 2015; Harris et al., 2016; Schaenzer et al., 2017; O’Malley et al., 2018).

In this study, the developed compounds were subjected to a phenotypic screening against various bacterial species including methicillin-sensitive as well as MRSA strains. This process identified a new class of molecules that were effective against MDR- *Staphylococcus aureus* as well as drug-tolerant MRSA persisters. Further studies indicated that these compounds rapidly eradicate MRSA bacteria through disrupting bacterial cell envelope. Additionally, MRSA did not develop resistance to these compounds during single-step or multistep resistance selection studies. The compounds did not cause significant hemolysis to human red blood cells. Toxicity studies have identified one compound, **6t**, with a high safety profile when tested in mice at high concentrations. Taken together, these molecules represent promising candidates for further investigation and development as treatment options against multidrug-resistant MRSA infections.

## Materials and Methods

### Bacterial Strains

A list of bacterial strains used in this study is presented in Supplementary Table 1. All strains were grown at 37°C in Mueller-Hinton (MH) media (Biolife, Italy) with the exception of *Enterococcus faecalis* which was grown in brain heart infusion (BHI) media (Biolife, Italy). Antibiotic resistance of bacterial strains was determined using agar disk diffusion assay according to Clinical and Laboratory Standards Institute guidelines (CLSI, 2018b). Strains resistant to at least three different classes of antibiotics are considered multidrug-resistance (Magiorakos et al., 2012). All MRSA strains exhibited resistance to cefoxitin (inhibition zone ≤ 21 mm), as a marker for the detection of MRSA (Fernandes et al., 2005).

### Synthesis of Compounds

The test compounds were synthesized according to our reported procedures (Al Tel et al., 2020; Srinivasulu et al., 2021). In brief, a solution of aldehyde (0.5 mmol) and 2-aminoazine (2/5, 0.5 mmol) in MeOH: DCM (1.5:0.5 ml) were added to a Ytterbium triflate (20 mol%) and sodium sulfate (1.0 mmol) solution at rt. After 45 min, tert-Butyl isocyanide (0.55 mmol) was added and stirring was continued for 12–15 h. After step-1 was completed verified by TLC analysis, an additional 30 mol% of Ytterbium triflate was added and the reaction was stirred at 70°C for another 12 h. Solvents were removed under vacuum and the crude was purified by flash column chromatography using EtOAc/hexane or MeOH/DCM as eluents to deliver pure products 4a-b/6a-y. The compounds characterization data including NMR and high resolution mass data were described in details in reference (Srinivasulu et al., 2021; Supplementary Material). Structures of the compounds are presented in Supplementary Table 2. Purity was > 95% as determined by HPLC and is presented for compounds **6s**, **6l**, and **6t** in Supplementary Figure 3.

***10-(4-Bromophenyl)-12-(tert-butylamino)-6-(2-methoxy-2- oxoethyl)-2,3,5,6,15,16-hexahydro-1H,14H-pyrido[3,2,1-ij]thi azolo[2′′,3′′:2′,3′]imidazo[1′,5′:4,5][1,4]oxazepino [7,6-f]quino lin-7-ium trifluoromethanesulfonate (6l).***

White solid, (199 mg, 51%). mp 178–180°C.

IR (neat): 3,329, 1,735, 1,504, 1,376, 1,275, 1,251, 1,161, 1,029 cm^–1^.

^1^H NMR (500 MHz, CD_3_OD) δ 7.74 (d, *J* = 8.3 Hz, 2H), 7.66 (d, *J* = 8.3 Hz, 2H), 7.56 (s, 1H), 7.29 (s, 1H), 5.22–5.14 (m, 1H), 4.65 (dd, *J* = 12.3, 4.4 Hz, 1H), 4.49 (dd, *J* = 12.3, 4.6 Hz, 1H), 3.66 (s, 3H), 3.31–3.22 (m, 4H), 3.04–2.91 (m, 2H), 2.84–2.72 (m, 4H), 2.02–1.94 (m, 4H), 0.58 (s, 9H).

^13^C NMR (125 MHz, CD_3_OD) δ 170.5, 150.4, 145.8, 144.4, 135.4, 133.5, 132.1, 131.1, 127.7, 127.3, 127.2, 124.0, 120.4 (q, *^1^J_*C*–*F*_* = 318.8 Hz), 117.3, 114.5, 112.1, 104.1, 72.7, 57.0, 54.9, 51.4, 49.4, 48.9, 34.1, 28.4, 26.9, 21.4, 20.8, 20.7. HRMS (ESI-TOF) m/z: [M-CF_3_SO_3_]^+^ calcd for: C_32_H_36_BrN_4_O_3_S; 635.1691 found: 635.1710.

***10-(4-Bromophenyl)-12-(tert-butylamino)-6-(2-methoxy-2- oxoethyl)-9-methyl-2,3,5,6,15,16-hexahydro-1H,14H-pyrido[3, 2,1-ij]thiazolo[2′′,3′′:2′,3′]imidazo [1′,5′:4,5][1,4]oxazepino [7,6-f]quinolin-7-ium trifluoromethanesulfonate (6s).***

Yellowish solid (231 mg, 58%). mp 200–202°C.

^1^H NMR (500 MHz, CD_3_OD) δ 7.77 (d, *J* = 8.5 Hz, 2H), 7.55 (d, *J* = 8.4 Hz, 2H), 7.26 (s, 1H), 5.17–5.09 (m, 1H), 4.63 (dd, *J* = 12.3, 4.5 Hz, 1H), 4.47 (dd, *J* = 12.3, 4.7 Hz, 1H), 3.75 (s, 0.5H), 3.67 (s, 3H), 3.29–3.21 (m, 4H), 3.03–2.88 (m, 2H), 2.82–2.72 (m, 4H), 2.47 (s, 3H), 2.07–1.90 (m, 4H), 0.56 (s, 9H).

^13^C NMR (125 MHz, CD_3_OD) δ 170.5, 150.3, 145.7, 141.5, 133.2, 132.6, 131.3, 130.1, 127.6, 127.4, 126.9, 126.2, 123.9, 120.4 (q, *^1^J_*C*–*F*_* = 320.0 Hz), 117.2, 112.0, 104.1, 72.7, 56.9, 54.7, 51.4, 49.4, 48.9, 34.1, 28.5, 26.9, 21.5, 20.8, 20.7, 11.6.

HRMS (ESI-TOF) m/z: [M-CF_3_SO_3_]^+^ calcd for: C_33_H_38_BrN_4_O_3_S; 649.1847 found: 649.1848.

***10-(Benzo[d][1,3]dioxol-4-yl)-12-(tert-butylamino)-6-(2- methoxy-2-oxoethyl)-9-methyl-2,3,5,6,15,16-hexahydro-1H,14 H-pyrido[3,2,1-ij]thiazolo[2′′,3′′:2′,3′]imidazo[1′,5′:4,5] [1,4] oxazepino[7,6-f]quinolin-7-ium trifluoromethanesulfo nate (6t).***

Yellowish solid (194 mg, 51%). mp 174–176°C.

IR (neat): 2,939, 1,738, 1,487, 1,446, 1,256, 1,201, 1,150, 1,028 cm^–1^.

^1^H NMR (500 MHz, CD_3_OD) δ 7.30 (s, 1H), 7.13 (d, *J* = 1.5 Hz, 1H), 7.11–7.03 (m, 2H), 6.14–6.07 (m, 2H), 5.15–5.08 (m, 1H), 4.62 (dd, *J* = 12.3, 4.0 Hz, 1H), 4.48 (dd, *J* = 12.3, 4.7 Hz, 1H), 3.67 (s, 3H), 3.28–3.20 (m, 4H), 3.03–2.86 (m, 2H), 2.80–2.73 (m, 4H), 2.46 (s, 3H), 2.03–1.92 (m, 4H), 0.62 (s, 9H). ^13^C NMR (125 MHz, CD_3_OD) δ 170.5, 150.3, 149.4, 148.0, 145.6, 141.4, 131.8, 131.0, 127.7, 127.5, 126.0, 125.5, 120.4 (q, *^1^J_*C*–*F*_* = 320.0 Hz), 120.3, 117.1, 111.9, 111.4, 107.9, 104.1, 101.9, 72.6, 57.0, 54.8, 51.4, 49.4, 48.9, 34.0, 28.6, 26.9, 21.5, 20.8, 20.7, 11.6.

HRMS (ESI-TOF) m/z: [M-CF_3_SO_3_]^+^ calcd for: C_34_H_39_N_4_O_5_S; 615.2641 found: 615.2659.

### Minimum Inhibitory Concentrations

MICs of the compounds were determined using twofold dilutions of compounds by broth microdilution method according to Clinical and Laboratory Standards Institute guidelines (CLSI, 2018a). Fresh bacteria grown on MH agar plates were harvested and adjusted to 0.5 McFarland (10^8^ CFU/ml) in sterile 0.85% NaCl solution. Bacterial suspensions were then diluted by 1:100 in MH broth media containing twofold dilutions of test compounds or antibiotics in sterile 96 well plates. The plates were then incubated aerobically at 37°C for 20 h and growth within the wells was determined visually.

### Time-Kill Study

Time-kill assay was performed by the broth microdilution method on 5 × 10^5^ CFU/ml exponentially grown bacterial cells exposed to twofold serial dilutions of test compounds (3.125–100 μg/ml). Viability was assessed by removing a sample at indicated time points followed by serial dilution (1:10 intervals) in 0.85% NaCl solution and plating 10–100 μl of each dilution onto MH agar plates. Plates were incubated at 37°C for 24 h (Mojsoska et al., 2017) and colonies were counted. All experiments were conducted in triplicate.

### Assay for Killing Methicillin-Resistant *Staphylococcus aureus* Persister Cells

Persister *S. aureus* was isolated by treating stationary phase antibiotic-susceptible bacteria with high antibiotic concentrations for 4 h (Kim et al., 2016, 2018c). The MRSA-3 strain was used in the persister experiments because this strain is susceptible to the antibiotics vancomycin, ciprofloxacin and gentamicin. MRSA-3 was grown in 25 ml MH broth for 16 h at 37°C, with shaking at 225 rpm to obtain stationary phase cells. Stationary phase cultures were washed 3 times with the same volume of phosphate buffered saline (PBS), and treated with 100 × MIC gentamicin for 4 h at 37°C, with shaking at 225 rpm. Bacteria were then washed 3 times with PBS, adjusted to 10^7^ CFU/ml and then treated with the test compounds or control antibiotics at indicated concentrations. At specific time points a 50 μl sample was removed, serially diluted, and spot-plated on MH agar plates to determine viable cell counts (Kim et al., 2015). The frequency of persister cells among stationary phase cultures of MRSA-3 are similar to previous observations (Allison et al., 2011; Ling et al., 2015; Kim et al., 2016, 2018c). The detection limit was 1:200 CFU/ml. A control experiment using actively growing MRSA-3 cells treated by 100 × MIC gentamicin was conducted to confirm that 100 × MIC gentamicin can eradicate none persister cells. Detection limit was 1:1,000 CFU/ml. Experiments were conducted on three biologically independent cultures.

### Fluorescence Microscopy

Bacteria were grown in MH broth to early logarithmic phase (2 × 10^5^ CFU/ml) then 2 ml cultures were treated 50 μg/ml of compounds or antibiotic and incubated at 37°C. At different intervals, cells were collected by centrifugation at 3,000 × g for 5 min and washed twice with sterile water. Bacterial pellets were then suspended in 50 μl of water and stained with the live and dead nucleic acid stains (Molecular Probes, United States): SYTO 9 (5 μM) and propidium iodide (15 μM) for 15 min at room temperature in the dark. 5 μl of the stained bacteria were placed on a sterile glass slide covered with a glass coverslip and imaged using fluorescence microscope (IX73, Olympus, United States) with excitation/emission of 480/500 nm for the green SYTO 9 and 490/635 nm for the red propidium iodide (Asadishad et al., 2011; Huang and Yousef, 2014). Numbers of red-fluorescing or yellow-fluorescing (dead) and green-fluorescing (live) cells were quantified with ImageJ software (National Institutes of Health, United States).

### Atomic Force Microscopy

Exponentially growing bacteria were adjusted to 10^8^ CFU/ml in MH broth and treated with 50 μg/ml of the test compounds or meropenem (50 μg/ml) for 4 h at 37°C. This concertation was chosen to account for the large number of cells needed in this procedure and is equivalate to 8 × MIC for **6l**, 16 × MIC for **6s,** and 25 × MIC for meropenem. Meropenem was used at highest fold MIC to maximize the likelihood of detecting any cell envelope disruption within 4 h. Cells were then centrifuged at 3,000 × g for 5 min, washed twice with sterile molecular biology grade water (Sigma-Aldrich, United States), then resuspended in 50 μl molecular biology grade water (Soon et al., 2009). 5 μl of concentrated bacterial suspension was spread on the surface of poly-L-lysine coated glass slide and air dried for 15 min. Slides were washed with sterile water to remove unattached bacterial cells and then air-dried. Slides were immediately examined under atomic force microscopy (NanoScience Nanosurf EasyScan 2 Flex AFM, Switzerland) (López-Jiménez et al., 2015). Images were acquired by tapping mode in air with a scan rate of 0.5–1 Hz at a resolution of 512 pixels per line (Soon et al., 2009). All experiments were conducted in triplicate.

### Single-Step Resistance Selection

Exponentially growing bacterial cells were adjusted to (∼10^10^ CFU/ml) and spread onto the surface of MH Agar plates containing twofold serial dilution of the test compounds (6.25–50 μg/ml). The plates were incubated at 37°C, and monitored for any growth over a duration of 72 h. The frequency of spontaneous bacterial resistance was calculated as the number of resistant colonies per inoculum. The frequency of spontaneous bacterial resistance was considered less than 1 in 10^10^ if no resistant colonies were observed at the end of the incubation period (Ling et al., 2015).

### Multi-Step Resistance Selection

Bacterial cells (5 × 10^5^ CFU/ml) were exposed to sub-MIC levels of the test compounds or ciprofloxacin diluted in 100 μl MH broth in a 96 well-plate. Bacterial cultures were incubated at 37°C for 24 h. Following incubation, aliquots from the well containing the highest concentration of test compound that permitted visible bacterial growth were removed and used to inoculate the next passage at 1:200 dilution. For each sequential passage, the concentration of the tested compound or antibiotic concentration was increased by 10% increments (up to 50% increase). This procedure was performed 24 consecutive times and conducted in triplicate (Lahiri and Alm, 2016).

### Toxicity to Red Blood Cells (*in vitro* Hemolysis)

Fresh human blood (group O +) was obtained from a healthy volunteer. The blood was centrifuged at 5,000 rpm for 5 min. Plasma and buffy coat were removed and red blood cells (RBCs) were prepared at 2% RBC suspension in 0.85% NaCl solution (Sharma and Sharma, 2001). Fresh RBC suspensions were treated with compounds at different concentrations in triplicates. A blank containing untreated RBC suspension was used as a negative control. 0.1% DMSO was used as a vehicle control and 0.1% Triton × 100 was used as a positive control. After 1 h of incubation at 37°C, cells were centrifuged, and the supernatant was transferred into flat bottom microtiter plate and used to measure the absorbance of the liberated hemoglobin at a wavelength of 550 nm. Hemolysis percentage for each sample was calculated by dividing sample’s absorbance on positive control absorbance (complete hemolysis) multiplied by 100. Negative control absorbance was subtracted from both samples and positive control absorbance before calculation (Joglekar et al., 2013). The experiment was conducted in triplicate.

### Animals

Adult SJL/J mice weighting 18–25 g were obtained from The Jackson Laboratory (Bar Harbor, ME) and housed in Sharjah Institute for Medical Research, University of Sharjah at constant temperature (25 ± 2°C), humidity (60 ± 10%) and a 12/12 h light/dark cycle. The mice were provided with standard chow and water *ad libitum*. Additionally, animals were acclimatized for 7 days before starting the experimental work.

### Single Dose Toxicity Testing

To identify the highest dose of compound **6l** or **6t** that does not cause side effects or overt toxicity for the study duration. In the single-dose toxicity study, we started by the sighting study, which allowed the selection of the appropriate starting dose for the main study (van den Heuvel et al., 1987; Stallard and Whitehead, 1995). Compound **6l** or **6t** or vehicle (0.5% DMSO in PBS) was administered by intraperitoneal (i.p) injection in a sequential manner (escalating dose design). A total of three animals was used for each dose level investigated. There were no significant changes in the animals’ body weight or behavior compared to the control group. On the other hand, the mouse injected with compound **6l** (60 mg/kg) died in less than 24 h, which was confirmed by another mouse receiving the same dose. All the other groups and control animals survived for 7 days in the main study.

### Repeated Dose Toxicity Testing

In the repeated dose study, a new set of 9 mice were divided into 3 groups (*n* = 3). These animals received daily doses of vehicle or compound **6l** (10 and 20 mg/kg) or **6t** (50 and 100 mg/kg) through i.p. injection for 14 successive days. None of the animals that received these doses showed any significant signs of toxicity or changes in body weights over the 14 days of treatment. In addition, the animals did not show any significant reversibility, persistence, or delayed occurrence of toxic effects for 14 days post-treatment.

### Toxicopathological Evaluation

For further assessment of potential toxicities, all animals were sacrificed at the end of the repeated dose toxicity study, and blood samples were collected for the determinations of hematologic and biochemical parameters. In addition, the vital organs of mice were collected for histopathological analysis.

### Hematologic Parameters

DxH520 Hematology Analyzer (Beckman Coulter, United States) was used to determine the counts of red blood cells (RBC), white blood cells (WBC), neutrophils, lymphocytes, monocytes, eosinophils, basophils, and platelets, in addition to hematocrit, hemoglobin, mean cell volume (MCV), mean cell hemoglobin (MCH), mean cell hemoglobin concentration (MCHC), red blood cell distribution width (RDW), red cell distribution width standard deviation (RDW-SD), and mean platelet volume (MPV).

### Biochemical Parameters

The biochemical parameters were measured using Chemistry Autoanalyzer A1511 (Adaltis Pchem 1, Italy). The parameters evaluated were alanine aminotransferase (ALT), aspartate aminotransferase (AST), Gamma-glutamyl transferase (GGT), total bilirubin, total protein, albumin, creatinine, urea, uric acid, creatine kinase, glucose, triglyceride, and cholesterol.

### Histopathology Analysis

Lungs, liver, spleen, brain, heart, and kidneys of the mice from the control group and treatment groups (50 and 100 mg/kg per day) were excised, cleaned from the connective tissue and fat and weighed. The organs were fixed in 10% neutral buffered formalin and processed using Excelsior™ AS Tissue Processor (Thermo Fisher Scientific, United States). Paraffin-embedded tissue sections were cut at 3.0 μm using Thermo Fisher Scientific™ HM 355S Automatic Microtome (Thermo Fisher Scientific, United States) and placed onto Superfrost slides. The sections were stained with hematoxylin and eosin following standard procedures. Photographs were taken using an Olympus microscope BX43 (Olympus, United States).

### Statistical Analysis

All statistical analyses were performed by PASW software version 18 (SPSS, Chicago, IL, United States). The data are reported as means ± standard deviation of the tested replicates. Statistical differences between control and treatment groups were analyzed by one-way ANOVA with *post hoc* Tukey test or using Kruskal–Wallis test for non-parametric analysis. All tests were two-tailed and a *p*-value < 0.05 was considered statistically significant.

## Results

### Identification of Antimicrobial Lead Candidates Effective Against Methicillin-Resistant *Staphylococcus aureus*

**FIGURE 1:**
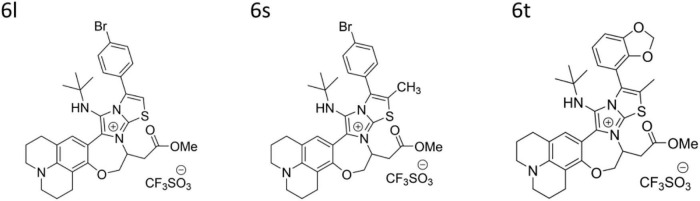
Chemical structures of compounds **6l**, **6s**, **6t**.

A pilot library of 29 polyheterocyclic compounds developed in our laboratory (Al Tel et al., 2020; Srinivasulu et al., 2021), were screened for their ability to inhibit the growth of Gram-positive *Staphylococcus aureus*, *Bacillus subtilis*, *Enterococcus faecalis*, and Gram-negative bacteria *Escherichia coli* and *Pseudomonas aeruginosa* (Supplementary Table 2). Of these, 17 compounds were active against the Gram-positive bacteria *Bacillus subtilis* and *Staphylococcus aureus* with minimum inhibitory concentration (MIC) values of 3.125–12.5 μg/ml. Some compounds had weak antimicrobial activity against *Enterococcus faecalis* while none had significant activity against the Gram-negative bacteria *Escherichia coli* and *Pseudomonas aeruginosa* (Supplementary Table 2). We next screened the active compounds against 10 MDR *Staphylococcus* bacteria including three clinical MRSA isolates and seven environmental *S. saprophyticus*, *S. epidermidis*, and *S. haemolyticus* isolates (Table 1). The MIC values for the most potent compounds on three MRSA strains, ranged from 3.125 to 6.25 μg/ml (Table 1). As a result, three of the most potent antibacterial compounds **6l**, **6s**, and **6t** were chosen for further investigation (Table 1 and Figure 1). These compounds, **6l**, **6s**, and **6t**, possess common functional groups, namely bromophenyl or dioxyphenyl truncated on imidazothiazole fused to benzoxazepine core scaffold. Deviation from this substitution patterns compromised their broad antibacterial activity (Figure 1 and Supplementary Table 2).

**TABLE 1:** MICs (μg/ml) for compounds tested on 10 multidrug-resistant strains of the genus *Staphylococcus*.

Compound ID	Clinical strains (*n* = 3)			Environmental strains (*n* = 7)							Summary of the 10 strains
			
	MRSA-1	MRSA-2	MRSA-3	UDH-1	UDH-2	UDH-3	UDH-4	UDH-5	UDH-6	UDH-7	MIC range
6f	12.5	12.5	12.5	25	12.5	12.5	12.5	6.25	25	25	6.25–25
6g	6.25	6.25	6.25	12.5	6.25	6.25	6.25	3.125	12.5	25	3.125–25
6j	6.25	6.25	3.125	6.25	6.25	3.125	6.25	1.56	6.25	12.5	1.56–12.5
6k	6.25	6.25	6.25	12.5	25	12.5	6.25	3.125	25	25	3.125–25
6l	**6.25**	**6.25**	**6.25**	**6.25**	**6.25**	**12.5**	**3.125**	**3.125**	**12.5**	**12.5**	**3.125–12.5**
6m	6.25	3.125	3.125	6.25	6.25	6.25	6.25	3.125	12.5	12.5	3.125–12.5
6n	12.5	12.5	12.5	12.5	25	12.5	12.5	6.25	50	50	6.25–50
6o	6.25	6.25	6.25	12.5	25	6.25	6.25	1.56	12.5	12.5	1.56–25
6q	12.5	12.5	12.5	12.5	12.5	12.5	12.5	6.25	25	25	6.25–25
6r	6.25	6.25	6.25	6.25	6.25	6.25	3.125	1.56	6.25	12.5	1.56–12.5
6s	**3.125**	**3.125**	**3.125**	**6.25**	**6.25**	**3.125**	**6.25**	**3.125**	**6.25**	**6.25**	**3.125–6.25**
6t	**3.125**	**3.125**	**3.125**	**6.25**	**6.25**	**3.125**	**6.25**	**1.56**	**6.25**	**6.25**	**1.56–6.25**
6u	12.5	12.5	12.5	12.5	25	12.5	25	6.25	50	50	6.25–50
6v	6.25	3.125	3.125	6.25	6.25	3.125	6.25	1.56	6.25	12.5	1.56–12.5
6w	6.25	6.25	6.25	12.5	6.25	12.5	3.125	1.56	12.5	12.5	1.56–12.5
6x	12.5	12.5	12.5	12.5	25	12.5	25	6.25	50	50	6.25–50
8b	12.5	12.5	12.5	50	25	25	25	6.25	50	25	6.25–50
Amikacin	16	8	2	8	0.25	0.5	<0.25	<0.25	4	4	<0.25–16
Ciprofloxacin	32	16	2	64	64	64	64	<0.25	64	64	<0.25–64
Meropenem	2	4	1	8	4	16	8	0.5	8	64	0.5–64
Vancomycin	1	0.5	1	2	0.5	1	0.5	1	1	1	0.5–2
Gentamicin	50	25	0.625	ND	ND	ND	ND	ND	ND	ND	ND*^a^*

*^a^ND, Not determined, compounds **6l**, **6s**, and **6t** are highlighted.*

### Compounds Exert Fast Killing Activity Against Methicillin-Resistant *Staphylococcus aureus*

**FIGURE 2:**
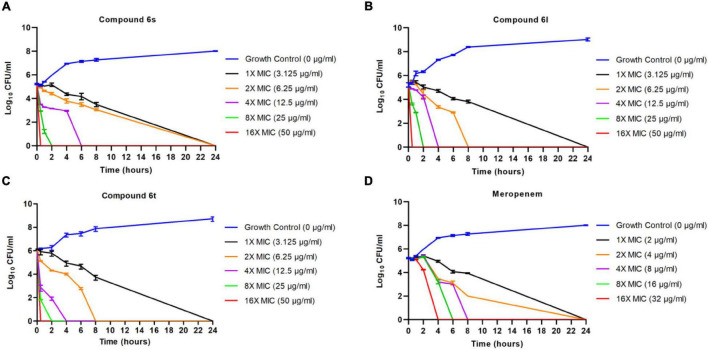
Killing kinetics of the compounds against exponentially growing cells: 10^6^ CFU/ml logarithmic phase MRSA-1 cultures were exposed to compounds **6l** **(A)**, **6s** **(B)**, **6t** **(C)**, or control antibiotic meropenem **(D)**. Viability was determined by serial dilution and CFU counts. Results are the average of 3 independent experiments.

We performed time-kill assays to study the killing kinetics of compounds **6s**, **6l**, and **6t** against exponentially growing (log phase) MRSA. At a concentration of 16 × MIC, compounds were able to rapidly eradicate exponentially growing MRSA-1 cells within 30 min (Figures 2A–C). The killing rate of these compounds was much faster than the control antibiotic meropenem which required 4 h to eradicate MRSA-1 bacteria at 16 × MIC (Figure 2D). All three compounds showed identical killing kinetics observed in MRSA-1 when tested against the other two clinical MRSA strains (MRSA-2 and MRSA-3) at 16 × MIC (Supplementary Figure 4).

### Compounds Cause Damage to Bacterial Cell Envelope

**FIGURE 3:**
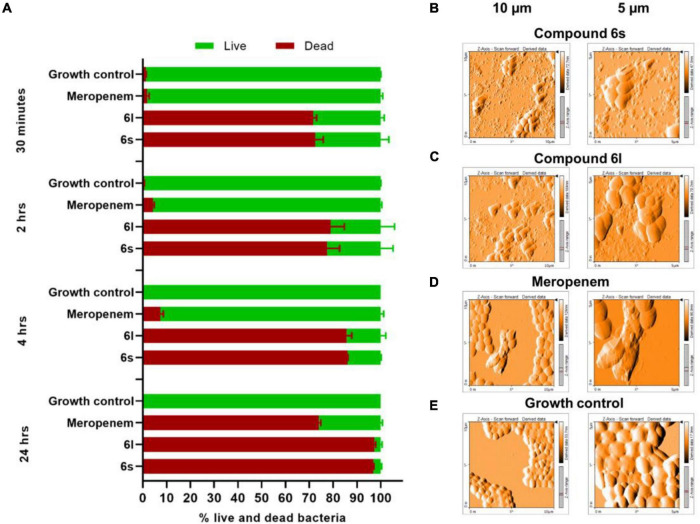
Microscopic examination of bacteria treated with test compounds: **(A)** Fluorescence microscope after staining with SYTO-9 (membrane permeable/green) and propidium iodide (membrane impermeable/red). MRSA-1 cultures (∼2 × 10^5^ CFU/ml) were treated with compounds **6s**, **6l** or control antibiotic meropenem for the indicated time. Results are the average of three independent experiments. Cells which stained green were counted as live whereases cell that stained red or yellow were counted as dead. **(B–E)** Atomic force microscopy of MRSA-1 (∼10^8^ CFU/ml) treated with compounds **6s** **(B)** and **6l** **(C)** or control antibiotic meropenem **(D)** at a concentration of 50 μg/ml for 4 h. Growth control **(E)** indicates untreated MRSA-1 culture.

We next sought to evaluate MRSA viability in response to compound treatment using dual staining with the fluorophores SYTO-9 and propidium iodide (PI). This approach differentiates live cells from dead cells by relying on membrane integrity as a proxy for cell viability (Berney et al., 2007; Stiefel et al., 2015). SYTO-9 (green fluorescent stain) is membrane permeable and stain cells independent of their membrane integrity. In contrast, PI (red fluorescent stain) can only penetrate bacteria with compromised membranes (Berney et al., 2007; Stiefel et al., 2015). Thus, live bacteria with intact membranes fluoresce green while dead cells with compromised membranes fluoresce red or yellow (Asadishad et al., 2011; Robertson et al., 2019). Exponentially growing cultures of MRSA-1 bacteria were treated with the test compounds **6s** and **6l**. After only 30 min of exposure to either compound, more than 70% of the MRSA bacteria stained red or yellow indicated a compromised cell membrane permeability and damage (Figure 3A and Supplementary Figure 5). This is in sharp contrast to the control antibiotic meropenem which had a much slower activity toward MRSA and was only able to achieve a comparable death rate of 74% upon extended treatment time of 24 h (Figure 3A). To further confirm these observations, atomic force microscope (AFM) was used to observe ultra-structural changes in bacterial cell envelopes in response to compound treatment. Treatment with compounds **6s** and **6l**, led to significant cell distortion and leakage of the cytoplasmic components indicating cell envelope damage (Figures 3B,C). On the other hand, meropenem-treated cells showed little distortion and leakage (Figure 3D) while untreated cells showed coccid cells indicating intact membrane (Figure 3E). Taken together, these observations demonstrate that these compounds can rapidly cause damage to bacterial cell envelopes.

### Compounds Eradicate Antibiotic-Tolerant Methicillin-Resistant *Staphylococcus aureus* Persisters

**FIGURE 4:**
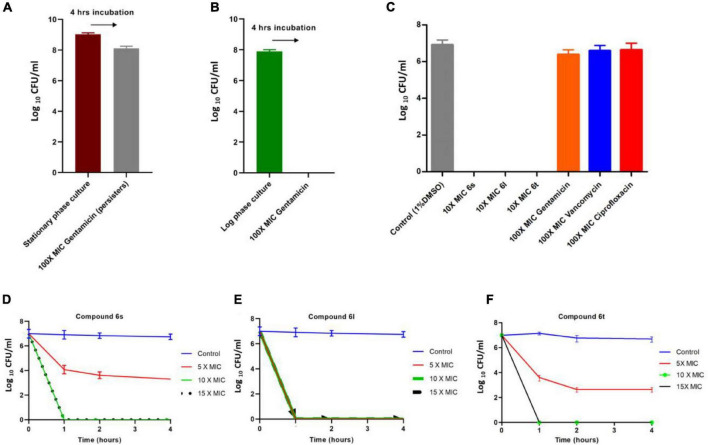
Killing kinetics of compounds against persister MRSA cells: **(A)** Development of MRSA-3 persister cells. Stationary phase overnight cultures of MRSA-3 were treated with 100 × MIC (62.5μg/ml) gentamicin for 4 h and viable drug tolerant persister counts were determined. **(B)** Gentamicin killing of log phase MRSA-3 cultures treated with 100 × MIC gentamicin for 4 h. **(C)** Persister MRSA-3 cells were diluted to 10^7^ CFU/ml and treated with indicated concentrations of compounds or control antibiotics for an additional 4 h. **(D–F)** Killing kinetics of compounds **6s (D)**, **6l** **(E)**, and **6t** **(F)** against persister MRSA-3 cells. All results are average of three biologically independent experiments.

The fast-killing rate and apparent lytic activity of test compounds suggest that they can be effective against metabolically inactive drug-tolerant MRSA persisters. *Staphylococcus aureus* develops high number of persisters cells during stationary phase and these persisters can be isolated by treating stationary phase antibiotic-susceptible bacteria with high antibiotic concentrations for 4 h (Lewis, 2010; Kim et al., 2015, 2018a,b,^2019^). To test the activity of compound against persisters MRSA, we used the MRSA-3 strain to generate presister cells since it is susceptible to the antibiotic gentamicin (Table 1). Treatment of stationary phase MRSA-3 cultures with 100 × MIC of gentamicin for 4 h resulted in little loss in cell viability (Figure 4A), in sharp contrast to the log phase MRSA-3 cultures which were completely eradicated by the same treatment (Figure 4B). When persister cells generated in Figure 4A were further treated for an additional 4 h with 100 × MIC of gentamicin, ciprofloxacin or vancomycin, no further loss in cell viability was observed (Figure 4C). On the other hand, compounds **6l**, **6s**, and **6t** completely eradicated the persister MRSA-3 after 4 h of treatment at a concentration of 10 × MIC (Figure 4C). MRSA-3 persisters were completely eradicated in a time-killing experiment after only 1 h of exposure to the compounds 5 × MIC for **6l** and 10 × MIC **6s** and **6t** (Figures 4D–F). These results indicate that the test compounds are highly active and can rapidly kill dormant and quiescent persister MRSA and offers further evidence that compounds damage cell membranes.

### Lack of Resistance Development by Methicillin-Resistant *Staphylococcus aureus* to Compounds

**FIGURE 5:**
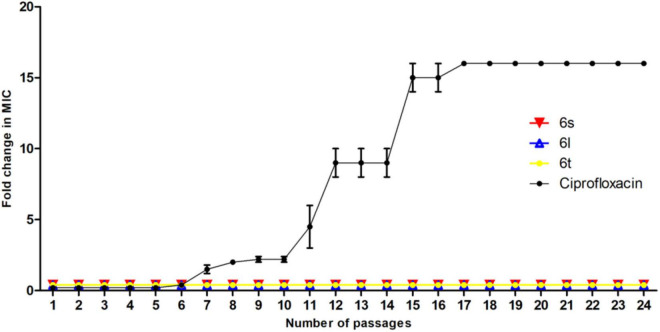
Multistep resistance during serial passaging in the presence of sub-MIC levels: MRSA-3 was grown at sub-MIC levels of test compound or control antibiotic ciprofloxacin. Cultures with positive growth at highest treatment concentration were used the next day to inoculate media at increasing increments of indicate compound. Experiment was conducted for 24 days and results are average of 3 independent experiments.

We evaluated the ability of MRSA to develop resistance to compounds **6s, 6l,** and **6t**. In single-step resistance selection experiments, no spontaneous resistant mutants were detected against compounds **6s, 6l,** and **6t**, indicating that the frequency for spontaneous resistance is less than 1 in 10^10^. Similarly, in multistep resistance selection studies, no mutants against **6s, 6l,** and **6t** emerged after 24 serial passages at sub-MIC levels, whereas serial passage in ciprofloxacin resulted in strains that were 16-folds more resistant than the parent MRSA strain (Figure 5). Taken together these results demonstrate that *S. aureus* can’t easily develop resistance antimicrobial action of these compounds.

### *In vitro* and *in vivo* Toxicity

**FIGURE 6:**
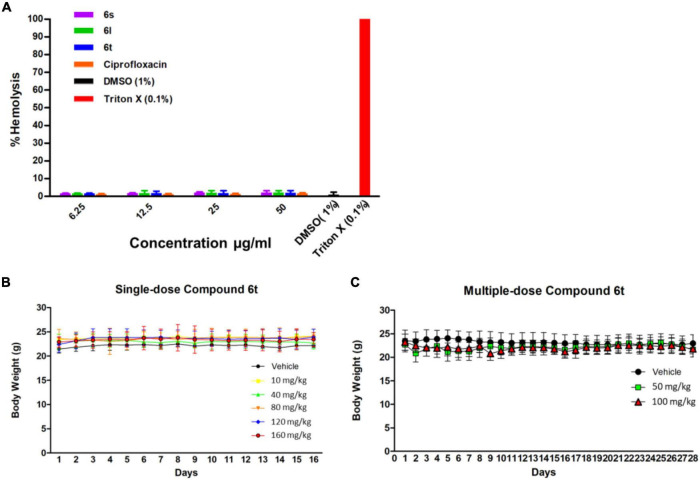
Toxicity assays. **(A)** Hemolysis assay for compounds and the control antibiotic ciprofloxacin at concentrations of 6.25–50 μg/ml. Triton × 100 (0.1%) was used as a positive control to produce complete hemolysis. **(B)** Effect of single-dose compound **6t** on mice weight over time. Mice were treated with indicated dose of **6t** vehicle (0.5% DMSO in PBS) and weight was monitor for 14 days (*n* = 3). **(C)** Effect of multiple-dose of compound **6t** on mice weight over time. Mice were treated with compound **6t** at concentration of 50 and 100 mg/kg/day or vehicle (0.5% DMSO in PBS) for 14 days and body weight was observed for an additional 14 days (*n* = 3).

To evaluate their safety profile, compounds **6l, 6s,** and **6t** were tested for their hemolytic activity against human red blood cells. Compounds were relatively safe when tested against human red blood cells, with less than 3% of the tested RBCs were hemolyzed in response to the test compounds even at a high concentration of 50 μg/ml (Figure 6A).

To further evaluate the safety profile *in vivo*, compound **6l** and **6t** was tested in adult SJL/J mice. Compound **6l** did not induce significant signs of toxicity, behavioral changes, or weight changes to mice after the administration of a single dose (10–40 mg/kg) or multiple-doses (10 and 20 mg/kg/day) treatments after 7 and 14 days observation, respectively (Supplementary Figure 6). However, at a single dose of 60 mg/kg the mice died with 24 h. On the other hand, compound 6t had an excellent safety profile with no signs of toxicity at up to 160 mg/kg (single-dose) and up to 100 mg/kg/day (multiple-dose) of 14 days successive treatment (Figures 6B,C). Animals receiving multiple-doses of **6t** were sacrificed after 2 weeks of the last dose and vital organs and whole blood were collected for histopathological and biochemical examination. Post-mortem inspection of lungs, liver, spleen, brain, kidney and heart indicated the absence of any gross lesions or significant changes in the organs size or weight (Supplementary Table 7). Moreover, microscopic examination did not reveal any histopathological changes or overt signs of toxicity in the organs of treated animals compared to the control group (Supplementary Figure 8). Comprehensive serum biochemical parameters were next performed on mice in response to repeated compound **6t** treatment. This was performed as an indicator of liver and kidney functions, heart and muscle damage, and metabolism. After 14 days of repeated treatment with compound **6t**, no significant changes of these serum biochemical parameters were detected compared to untreated controls (Supplementary Table 9). Similarly, hematological parameters, white blood cells, RBCs and platelets, were not affected by repeated treatment with compound **6t** when compared to untreated controls (Supplementary Table 9). Taken together, these results highlight the safety of compound **6t** in mice on both acute- and multiple-dose treatment protocols.

## Discussion

In this work, we evaluated the antimicrobial activity of a series of a pilot library of small compounds developed by our laboratory. As can be concluded from Supplementary Table 2 and after a series of structural optimization and biological activity evaluations, compounds containing imidazopyrazine, imidazoisoquinoline (compounds **4a**-**b**) are relatively not active against the tested bacterial strains. Trading imidazopyrazine with imidazobenzothiazole (compound **6a**) retains some of the antibacterial activity. However, replacing the benzene ring in **6a** with hydrogens as in **6b**, eliminates the activity. Trading the benzothiazole ring with di-substituted thiazole ring as in **6c**, retains the antibacterial activity. Substituting the bezooxazepine ring with fluorine atoms eliminates the activity (compound **6d**). However, fusing the benzooxazepine ring with another benzene ring as in compounds **6e**-**f** produces a relatively potent antibacterial activity. Positional variation of the new ring fusion improves the potency twofolds as in compound **6g**, whereas in compound **6h** the potency was lost. Interestingly, substituting the benzoxazepine ring with an amine group enhanced the antibacterial activity (compounds **6i**-**j**). Rigidifying the newly introduced amine group in rings (e.g., quinazoline ring) as in compounds **6l**-**m**) retains the antibacterial activity. With this data in hand, we screened various derivatives of compound **6k**, which identified a few compounds that displayed potent antibacterial activities. As a result, compounds containing a substituted diphenyl benzothiazole group anchored on a quinazoline ring fused to benzooxazepine core exhibited excellent antibacterial activity against Gram-positive bacteria. From this data, we concluded that a hybrid structure that encompasses a tetrahydro-1H,5H-pyrido[3,2,1-ij]quinolone fused to imidazo[2,1-b]thiazol-7-ium (compounds **6k**-**6y**) system is necessary for potent antimicrobial activity. However, these cationic benzooxazepine-based compounds displayed a poor antibacterial activity against Gram-negative bacteria. This could be due to the outer membrane permeability barrier of Gram-negative bacteria (Ghai and Ghai, 2018).

Infections caused by *Staphylococcus aureus* and MRSA represent a serious global health challenge (Turner et al., 2019). Treatment of such infections is often difficult due to bacterial resistance to antibiotics and the ability to form drug-tolerant biofilms and persister cells (Lewis, 2005). Studies aimed at eradicating drug-tolerant persister have led to the identification of two main strategies; through metabolic activation (Allison et al., 2011) or by targeting bacterial membranes (Kim et al., 2018a). Most of these strategies have focused on agents that target bacterial membrane since they are essential for cell viability but are independent of the cell’s low metabolic profile. Over the past few years, several membrane-active compounds that can eradicate *S. aureus* persisters have been identified and developed. These include repurposed antibiotics (Kim et al., 2019), investigational drugs (Kim et al., 2018b), and novel synthetic molecules (Kim et al., 2018c).

The fast-killing rate of the compounds (**6S**, **6l**, and **6t**) presented in this work against both actively growing and persister MRSA along with the microscopic analysis indicate antibacterial action through cell envelope disruption and bacterial lysis. This cell envelop damage could be a direct effect through damage to bacterial membranes or indirectly through damaging the peptidoglycan cell wall by inhibiting enzymes involved in cell wall synthesis and maintenance. Our data, the fast-killing rate and ability to kill persisters, support the notion that the antibacterial effects of compounds is mediated through membrane damage rather than cell wall damage. The compounds (**6S**, **6l**, and **6t**) were able to completely eradicate MRSA within only 30 min. This killing rate is much faster than that of meropenem which targets cell wall synthesis and requires a much longer time, 4 h, to achieve complete killing. The same fast rate of killing, 30 min, was also directly observed in the fluorescence microscopy experiment for compounds but not for meropenem treatment. Furthermore, the ability of compounds to eradicate persister MRSA, which also occurred at a fast rate, strongly suggests that the compounds target bacterial members rather than the cell walls. This is supported by the fact that almost all small compounds that kill metabolically inactive persisters by cell lysis do so by disrupting bacterial membranes rather than cell walls (Kim et al., 2018a). While molecules like lysostaphin and endolysin can kill MRSA persister by targeting bacterial cell walls, these are enzymes produced by phages and bacteria rather than small synthetic molecules (Bastos et al., 2010; Gutiérrez et al., 2014). Despite the fast killing kinetics of compounds against both actively dividing cells and persister cells, there was a more robust killing activity against dividing cells compared to persister cells. This is likely due to the difference in cell numbers between both experiments as well as the fundamental physiological, metabolic, and transcriptional difference between active and persister cells (Schilcher and Horswill, 2020). While membrane targeting molecules can kill both active and persister cells, the latter require longer treatment and higher concertation to achieve complete eradication (Mohamed et al., 2016).

The antimicrobial action of compounds (**6S**, **6l**, and **6t**) could be mediated by the interaction between the positively charged compounds and Gram-positive bacteria’s negatively charged cell envelope (Brunetti et al., 2016). Approximal 25% of Gram-positive bacterial membrane lipid bilayers contain anionic (negatively charged) phospholipids, whereas mammalian membranes are composed of zwitterionic (neutral) phospholipids and 20–50% cholesterol (Brender et al., 2012; Deleu et al., 2014). This is in line with our observations whereby the compounds (**6S**, **6l**, and **6t**) were highly active against MRSA but had a very little hemolytic effect against human red blood cells. This limited hemolytic activity observed is usually unavoidable among membrane targeting agents (Mohammad et al., 2015; Liu et al., 2017) and have also been observed with other recently discovered compounds that target persisters (Kim et al., 2016, 2018c). When compounds **6l** and **6t** were evaluated for their safety profiles in mice, compound **6t** had a superior safety profile with no observable side effects of treated mice both in short term and long-term treatments protocols even at high doses. The safety profile of compound **6t**, its fast killing activity against active and persister MRSA, and the inability of MRSA to develop resistance to its antimicrobial action makes it ideal for further investigation as a potential therapeutic against MRSA infection.

## Conclusion

We have identified new compounds that possess potent antibacterial activity against MDR *S. aureus* including MRSA persisters. These compounds cause rapid cell envelope damage leading to rapid killing of MRSA. Importantly, the compounds showed limited hemolytic activity against human RBCs and compound **6t** displayed an excellent safety profile in mice on both single- and multiple-dose treatments. Another important aspect of these compounds is their ability to rapidly eradicate drug-tolerant persister cells. Importantly, MRSA was not able to develop resistance toward these lead drug candidates after 24 serial passages compared to the significant resistance developed with conventional antibiotics. The unusual molecular skeleton and unique structural features of these motifs, that meet the charge, rigidity and shape requirements, together with the positive *in vitro* anti-MRSA results, highlight the importance for further studies to evaluate this new class of chemotypes in preclinical and clinical settings as potential antibacterial agents.

## Data Availability Statement

The original contributions presented in the study are included in the article/Supplementary Material, further inquiries can be directed to the corresponding author/s.

## Ethics Statement

The animal study was reviewed and approved by the number of animals, study design, and treatments protocol were reviewed and approved by the University of Sharjah Animal Care and Use Committee (Reference No. ACUC-18-12-17-1). All procedures in this protocol were firmly performed according to the Guide for the Care and Use of Laboratory Animals published by the US National Institute of Health (NIH publication No. 80-23, revised 1996).

## Author Contributions

FA-M, MH, and AS conducted the microbiology experiments under the supervision of MH. TA-T, MH, and FA-M wrote the first draft of the manuscript. VS synthesized the compounds and characterized the structures under the supervision of TA-T. TA-T conceived the idea and supervised all biological and chemical studies. FA-M and DZ performed the animal study, clinical chemistry, and histopathology experiment under supervision of HO. MH, GO, and TA-T corrected and finalized the manuscript which was submitted with the consent of all authors.

## Conflict of Interest

The authors declare that the research was conducted in the absence of any commercial or financial relationships that could be construed as a potential conflict of interest.

## Publisher’s Note

All claims expressed in this article are solely those of the authors and do not necessarily represent those of their affiliated organizations, or those of the publisher, the editors and the reviewers. Any product that may be evaluated in this article, or claim that may be made by its manufacturer, is not guaranteed or endorsed by the publisher.
