# Supplementary material for: Antibacterial Activity of Small Molecules Which Eradicate Methicillin-Resistant Staphylococcus aureus Persisters
Source: Front Microbiol. 2022 Feb 1;13:823394. doi: 10.3389/fmicb.2022.823394 (PMC8846302; doi:10.3389/fmicb.2022.823394)
Supplement: Supplementary file 1 [file Data_Sheet_1.docx]

**Supplementary Material**

**Antibacterial activity of small molecules which eradicate methicillin-resistant *Staphylococcus aureus* (MRSA) persisters**

**Mohamad Hamad^1,2^, Farah Al-Marzooq^1,3^, Vunnam Srinivasulu ^1^, Hany A. Omar ^1,4^, Ashna Sulaiman^1^, Dana M. Zaher ^1^, Gorka Orive^5^ and Taleb H. Al-Tel ^1,4*^**

^1^Sharjah Institute for Medical Research, University of Sharjah, Sharjah, United Arab Emirates

^2^ College of Health Sciences, University of Sharjah, Sharjah, United Arab Emirates

^3^College of Medicine and Health Sciences, UAE University, Al Ain, United Arab Emirates

^4^College of Pharmacy, University of Sharjah, Sharjah, United Arab Emirates

^5^ NanoBioCel Group, Laboratory of Pharmaceutics, School of Pharmacy, University of the Basque Country (UPV/EHU), Paseo de la Universidad 7, 01006 Vitoria-Gasteiz, Spain

* Corresponding author

E-mail address: [taltal@sharjah.ac.ae](mailto:taltal@sharjah.ac.ae)

College of Pharmacy and Sharjah Institute for Medical Research

University of Sharjah, P.O. Box 27272, Sharjah, UAE

**List of contents:**

1. List of bacterial strains used in this study.
2. Structure of compounds and their Minimum Inhibitory Concentrations (MICs).
3. HPLC data of compounds **6s**, **6l**, **6t**.
4. Killing kinetics of the compounds against exponentially growing MRSA strains.
5. Fluorescence microscopy images of bacteria treated with test compounds for 30 minutes.
6. Toxicity effects of effects of compound **6l** on mice weight over time.
7. Relative organ weight in mice treated with repeated dose of compound **6t** for 14 days.
8. Histopathology-based toxicity studies for compound **6t**.
9. Clinical chemistry and haematology parameters in mice treated for 14 days with repeated dose of compound **6t**.
10. Compounds characterization data

**Supplementary Table 1S. List of bacterial strains used in this study**

| **Organism (strain)** | **Source** | **Relevant characteristic/phenotype** |
| --- | --- | --- |
| *Escherichia coli* (ATCC 25922) | Reference strain (ATCC) | Gram negative bacteria |
| *Pseudomonas aeruginosa* (ATCC 27853) | Reference strain (ATCC) | Gram negative bacteria |
| *Enterococcus faecalis* (ATCC 29212) | Reference strain (ATCC) | Gram positive bacteria |
| *Bacillus subtilis* (BS1) | Environmental isolate | Gram positive bacteria |
| *Staphylococcus aureus* (ATCC 25923) | Reference strain (ATCC) | Gram positive bacteria |
| *S. aureus* (MRSA-1*) | Clinical isolate | MDR # (COT, CPD, CCL,GEN, CIP, CX) |
| *S. aureus* (MRSA-2*) | Clinical isolate | MDR (COT, CPD, CCL,GEN CIP, CX) |
| *S. aureus* (MRSA-3*) | Clinical isolate | MDR (CPD, CCL, CX) |
| *S. saprophyticus* (UDH-1) | Environmental isolate | MDR (COT, CPD, CCL, CIP, CX) |
| *S. saprophyticus* (UDH-2) | Environmental isolate | MDR (COT, CPD, CCL, CIP, CX) |
| *S. saprophyticus* (UDH-3) | Environmental isolate | MDR (COT, CPD, CCL, CIP, CX, MRP) |
| *S. saprophyticus* (UDH-4) | Environmental isolate | MDR (COT, CPD, CCL, CIP, CX) |
| *S. epidermidis* (UDH-5) | Environmental isolate | MDR (CTR, CPD, CCL, CPM, CX) |
| *S. haemolyticus* (UDH-6) | Environmental isolate | MDR (CPD, CCL, CIP, CX) |
| *S. haemolyticus* (UDH-7) | Environmental isolate | MDR (CPD, CCL, CIP, CX, MRP) |

* Methicillin-resistant *Staphylococcus aureus* (MRSA)

# MDR: Multidrug resistant. Antibiotic sensitivity testing was done using agar diffusion method with the following antibiotic disks (Himedia, India): CTR (ceftriaxone; 30 µg), CX (cefoxitin; 30 µg), CPM (cefepime; 30 µg), CPD (cefpodoxime; 10 µg), CCL (cefpodoxime/ clavulanic acid ; 10 µg/5 µg), MRP (meropenem; 10 µg), CIP (ciprofloxacin; 30 µg), COT (trimethoprim/sulphamethoxazole; 1.25/23.75 µg) and GEN (gentamicin; 50 µg).

**Supplementary Table S2. Chemical structure of compounds and their minimum inhibitory concentrations against a series of bacterial strains.**

| **Compound** | **Structure** | **Bacterial species** | | | | | |
| --- | --- | --- | --- | --- | --- | --- | --- |
|  |  | **Gram-positive bacteria** | | | | **Gram-negative bacteria** | |
|  |  | ***Staphylococcus***  ***aureus***  ATCC 25923 | ***Bacillus***  ***subtilis*** | ***Enterococcus***  ***faecalis***  ATCC 29212 | ***Escherichia***  ***coli***  ATCC 25922 | | ***Pseudomonas***  ***aeruginosa***  ATCC 27853 |
| **4a** |  | >100 | >100 | Non-active | Non-active | | Non-active |
| **4b** |  | 50 | 100 | >100 | Non-active | | Non-active |
| **6a** |  | 25 | 50 | Non-active | Non-active | | Non-active |
| **6b** |  | >100 | >100 | >100 | Non-active | | Non-active |
| **6c** |  | 25 | 25 | >100 | Non-active | | Non-active |
| **6d** |  | >100 | >100 | Non-active | Non-active | | Non-active |
| **6e** |  | 25 | 12.5 | 100 | Non-active | | Non-active |
| **6f** |  | 12.5 | 12.5 | 25 | >200 | | Non-active |
| **6g** |  | 6.25 | 3.125 | 50 | >200 | | Non-active |
| **6h** |  | 100 | 100 | 400 | Non-active | | Non-active |
| **6i** |  | 12.5 | 6.25 | 50 | 200 | | Non-active |
| **6j** |  | 6.25 | 3.125 | 100 | Non-active | | Non-active |
| **6k** |  | 6.25 | 12.5 | 50 | Non-active | | Non-active |
| **6l** |  | 3.125 | 6.25 | 50 | Non-active | | Non-active |
| **6m** |  | 3.125 | 3.125 | 25 | Non-active | | Non-active |
| **6n** |  | 6.25 | 12.5 | 100 | Non-active | | Non-active |
| **6o** |  | 3.125 | 3.125 | 50 | Non-active | | Non-active |
| **6p** |  | 12.5 | 12.5 | 25 | Non-active | | Non-active |
| **6q** |  | 6.25 | 3.125 | 100 | Non-active | | Non-active |
| **6r** |  | 3.125 | 3.125 | 50 | 200 | | Non-active |
| **6s** |  | 3.125 | 3.125 | 25 | 200 | | Non-active |
| **6t** |  | 3.125 | 3.125 | 25 | Non-active | | Non-active |
| **6u** |  | 12.5 | 25 | 100 | Non-active | | Non-active |
| **6v** |  | 6.25 | 3.125 | 100 | Non-active | | Non-active |
| **6w** |  | 3.125 | 3.125 | 50 | Non-active | | Non-active |
| **6x** |  | 12.5 | 50 | >100 | Non-active | | Non-active |
| **6y** |  | >100 | >100 | Non-active | Non-active | | Non-active |
| **8a** |  | 100 | 100 | >100 | Non-active | | Non-active |
| **8b** |  | 12.5 | 6.25 | 25 | >200 | | Non-active |
| Amikacin |  | 4 | 0.5 | 256 | 1 | | 1 |
| Ciprofloxacin |  | 0.125 | 0.05 | 0.5 | 0.008 | | 0.25 |


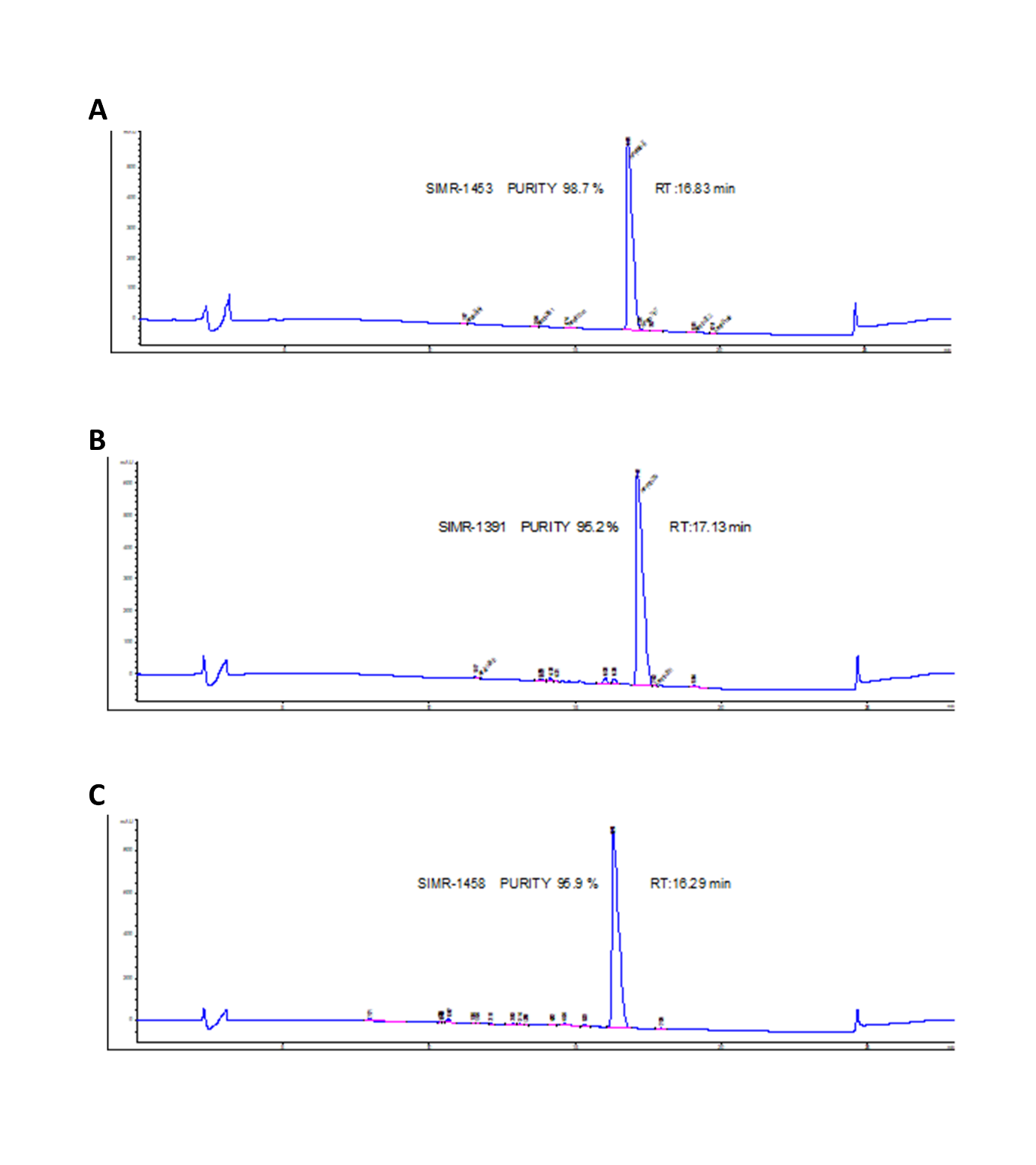


**Supplementary Figure 3S.** HPLC chromatogram of compounds (**A**) **6l**, (**B**) **6s**, (**C**) **6t**.

**
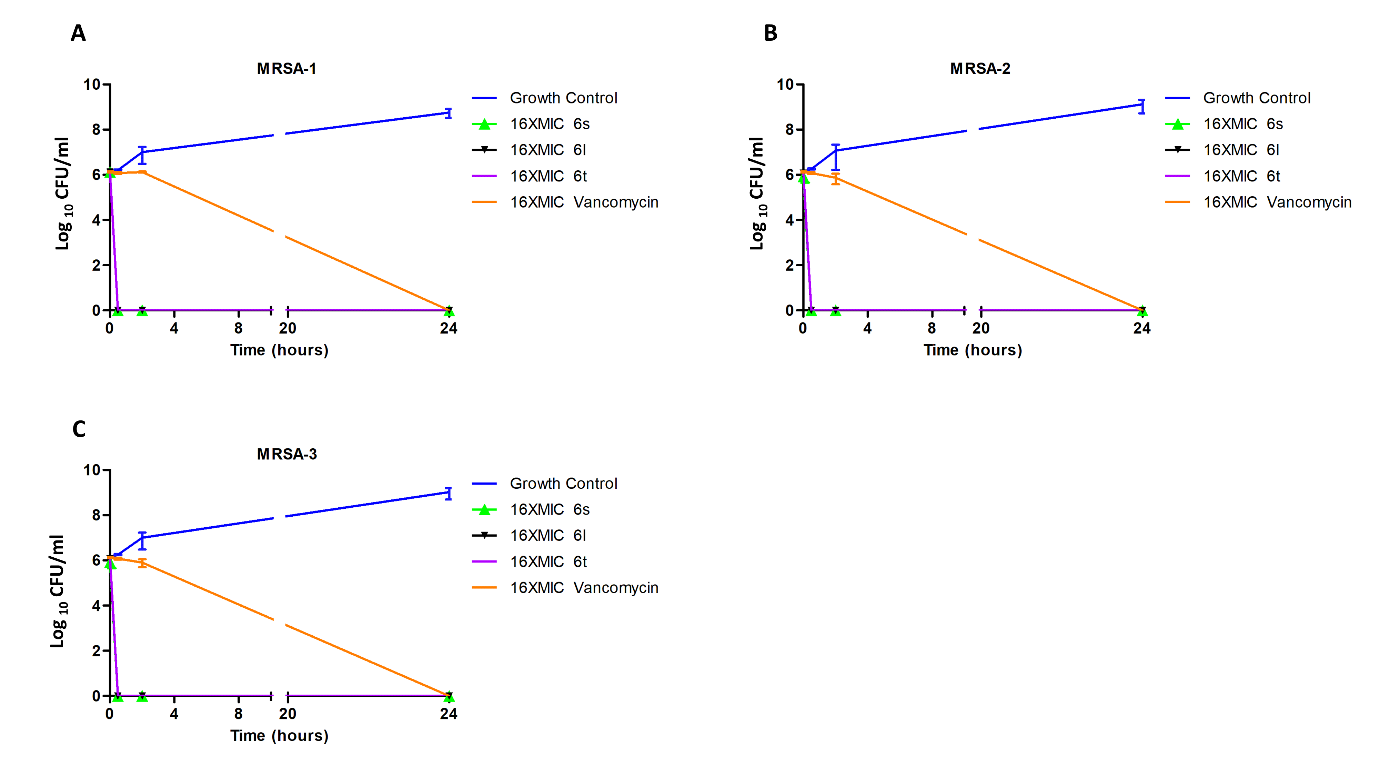
**

**Supplementary Figure 4S.** Killing kinetics of the compounds against exponentially growing cells of MRSA-1 (**A**), MRSA-2 (**B**), and MRSA-3 (**C**). 10^-6^ CFU/ml logarithmic phase cultures were exposed to compounds **6s, 6l,** and **6t** or control antibiotic vancomycin. Viability was determined by serial dilution and CFU counts after 30 min, 2 hours and 24 hours of treament with the compounds. Results are the avarage of 3 independent experiments.


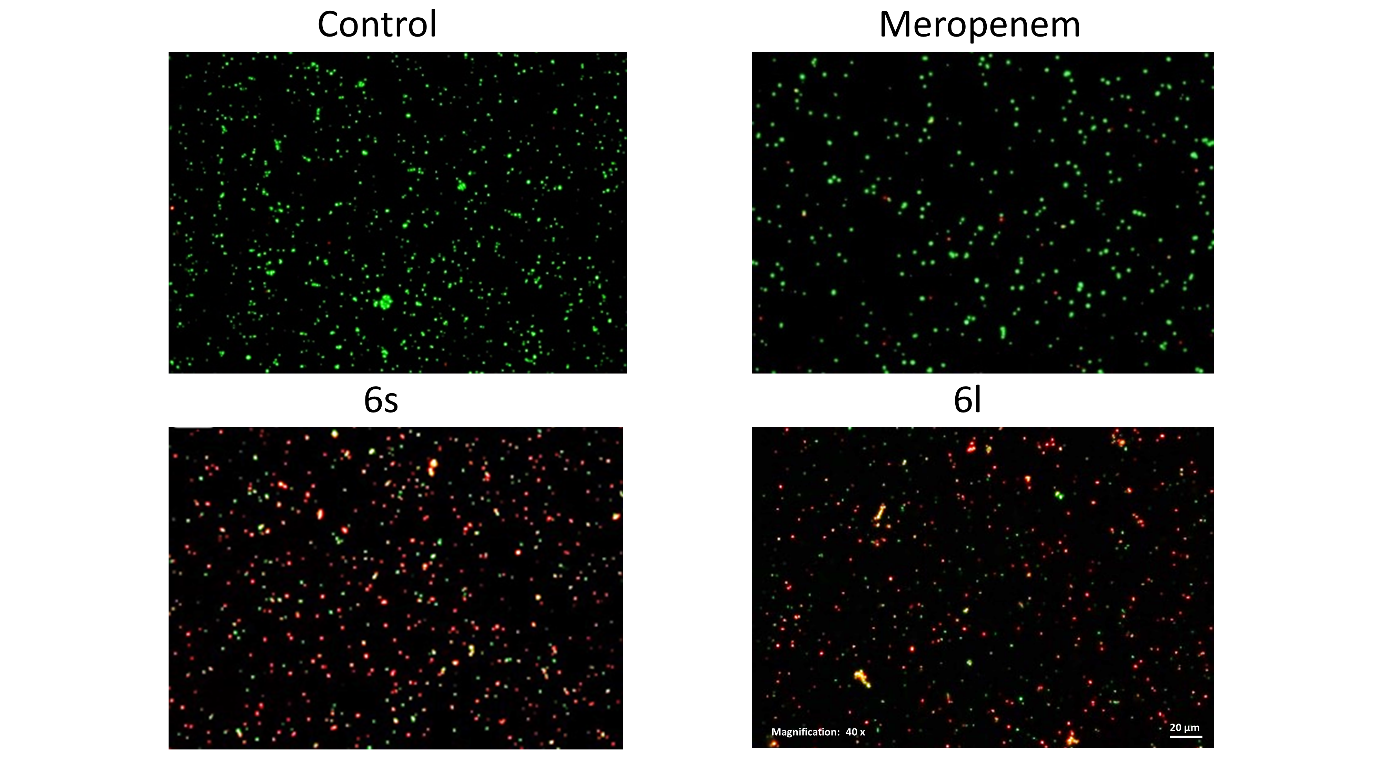


**Supplementary Figure 5S.** **Microscopic examination of bacteria treated with test compounds for 30 minutes.** Fluorescence microscopy images of MRSA-1 stained with SYTO-9 (membrane permeable/green) and propidium iodide (membrane impermeable/red). MRSA-1 cultures (~2 ×10^5^ CFU/ml) were treated with compounds **6s**, **6l** or control antibiotic meropenem for 30 minutes. Control indicates untreated culture.


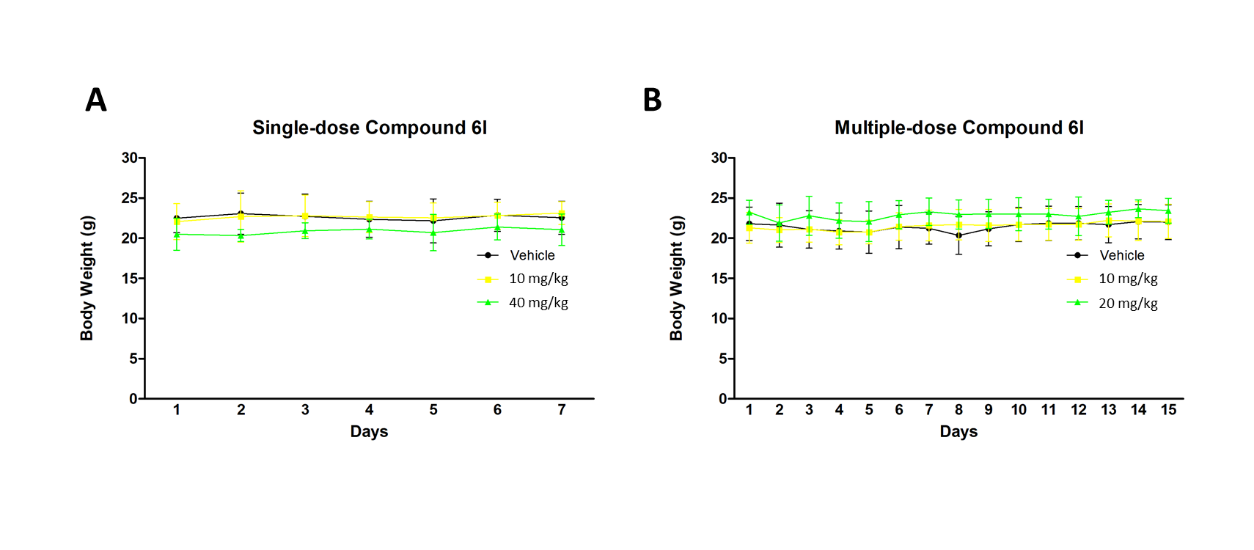


**Supplementary Figure 6S.** Effect of single (**A**) and multiple dose (**B**) administration of compound 6l on mice weight over time. (**A**) 7 days observation of the body weight of mice treated with a single dose compound **6l** at concentration of 10 mg/kg or 40 mg/kg or vehicle (0.5% DMSO in PBS) (n=3). (**B**) 14 days observation of the body weight of mice treated for 14 days with repeated doses of the compound **6l** at concentration of 10 mg/kg/day or 20 mg/kg/day or vehicle (0.5% DMSO in PBS) (n=3).

**Supplementary Table 7S**. Relative organ weight in mice treated for 14 days with repeated dose of compound **6t**. Mean ± standard deviation of the tested parameters from each group are shown

| **Treatment** | | **Mouse #** | **Relative Organ Weight (% of total body weight)** | | | | | | |
| --- | --- | --- | --- | --- | --- | --- | --- | --- | --- |
|  |  |  | **Brain** | **Heart** | **Liver** | **Left Kidney** | **Right Kidney** | **Spleen** | **Lung** |
| **Vehicle** | | A2 | 2.40 | 0.51 | 4.37 | 0.55 | 0.68 | 0.31 | 1.16 |
|  |  | A3 | 1.84 | 0.69 | 5.57 | 0.61 | 0.62 | 0.32 | 0.83 |
|  |  | A4 | 1.94 | 0.53 | 5.94 | 0.55 | 0.55 | 0.29 | 1.08 |
|  |  | Mean±SD | 2.06±0.3 | 0.58±0.1 | 5.30±0.82 | 0.57±0.04 | 0.62±0.06 | 0.31±0.01 | 1.02±0.17 |
| **Compound 6t** | **50 mg/kg/day** | B2 | 1.82 | 0.66 | 5.61 | 0.56 | 0.59 | 0.34 | 0.73 |
|  |  | B4 | 1.92 | 0.72 | 7.24 | 0.70 | 0.71 | 0.29 | 1.02 |
|  |  | B5 | 2.01 | 0.78 | 4.05 | 0.70 | 0.83 | 0.36 | 0.75 |
|  |  | Mean±SD | 1.92±0.09 | 0.72±0.06 | 5.63±1.59 | 0.65±0.08 | 0.71±0.12 | 0.33±0.03 | 0.83±0.16 |
|  | **100 mg/kg/day** | C2 | 1.99 | 0.69 | 4.51 | 0.72 | 0.75 | 0.36 | 0.67 |
|  |  | C3 | 2.07 | 0.68 | 4.68 | 0.73 | 0.77 | 0.29 | 1.03 |
|  |  | C5 | 2.15 | 0.58 | 4.20 | 0.65 | 0.68 | 0.33 | 0.65 |
|  |  | Mean±SD | 2.07±0.08 | 0.65±0.06 | 4.46±0.24 | 0.7±0.05 | 0.73±0.05 | 0.32±0.04 | 0.78±0.21 |
| ***p* value *** | | | 0.590 | 0.161 | 0.422 | 0.094 | 0.279 | 0.657 | 0.305 |

* Non-significant difference (p > 0.05)

# one-way ANOVA was used to compare the 3 groups.

.


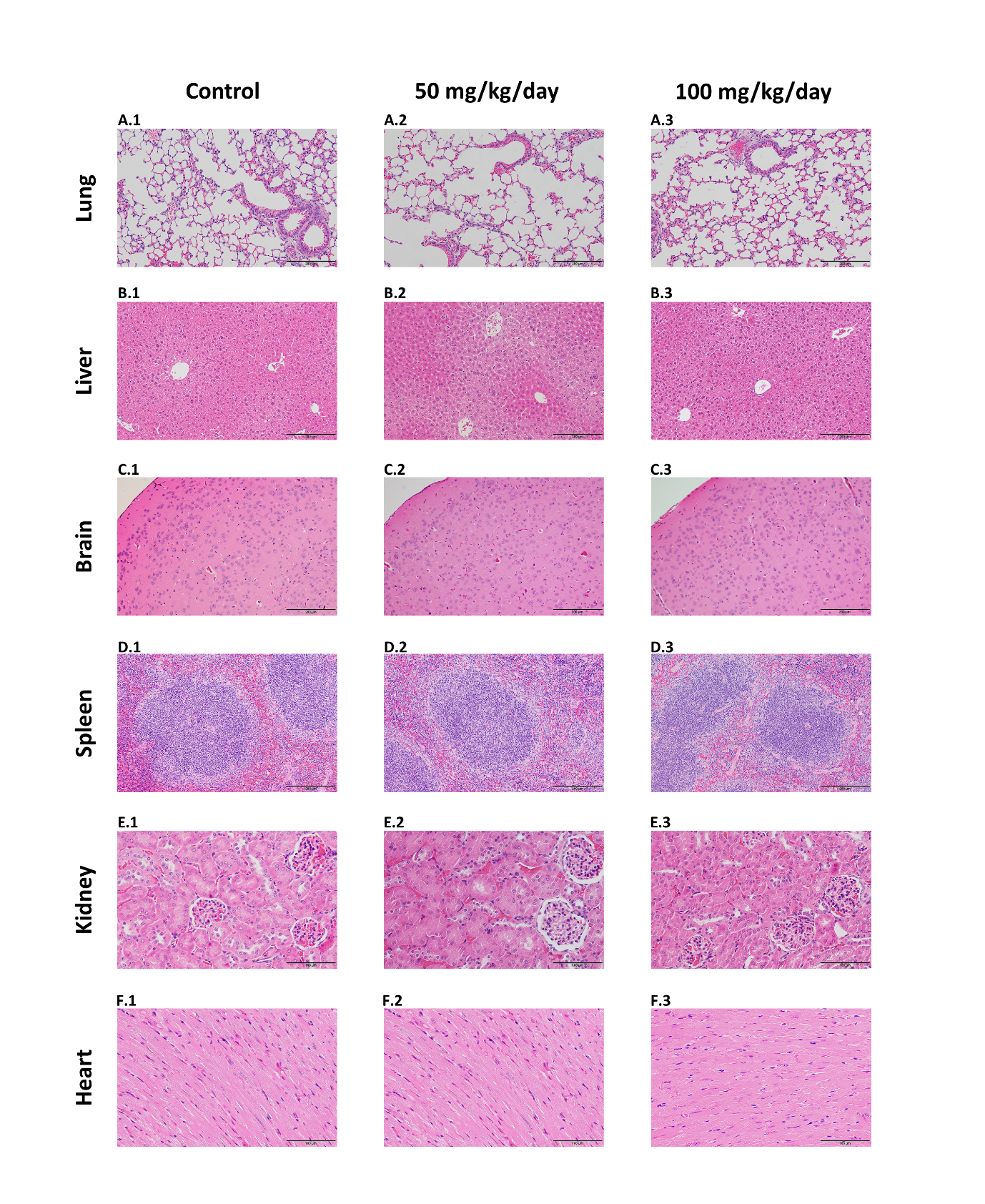


**Supplementary Figure 8S. Histopathology-based toxicity studies.** Images of (**A**) lung, (**B**) liver, (**C**) brain, (**D**) spleen, (**E**) kidney, (**F**) heart of mice treated with vehicle (control) and compound **6t** at a dose of 50 and 100 mg/kg/day, respectively. Hematoxylin-eosin stained sections of the organs from representative mice from each group are shown. Magnification, ×200 (**A-D**), Magnification, ×400 (**E-F**).

**Supplementary Table 9S.** Clinical chemistry and hematology parameters in mice treated for 14 days with repeated dose of compound **6t**. Mean ± standard deviation of the tested parameters from each group are shown

| **Treatment** | | **Vehicle** | **Compound 6t** | | ***p* value *** |
| --- | --- | --- | --- | --- | --- |
|  |  |  | **50 mg/kg/day** | **100 mg/kg/day** |  |
| **Number of mice** | | n=3 | n=3 | n=3 |  |
| **Clinical chemistry parameters** | | | | | |
| **Liver** | ALT (U/L) | 36.91±6.92 | 42.10±7.60 | 38.78±15.43 | 0.839 |
|  | AST (U/L) | 87.0±15.14 | 99.6±41.29 | 125.47±39.56 | 0.425 |
|  | GGT (U/L) | 0.38±0.07 | 0.40±0.07 | 0.34±0.06 | 0.502 |
|  | Total bilirubin (mg/dL) | 0.57±0.01 | 0.58±0.04 | 0.57±0.02 | 0.674 |
|  | Total protein (g/dL) | 5.21±0.23 | 5.27±0.17 | 5.20±0.14 | 0.881 |
|  | Albumin (g/dL) | 2.97±0.52 | 3.15±0.11 | 3.21±0.11 | 0.656 |
| **Kidney** | Creatinine (mg/dL) | 0.76±0.03 | 0.8±0.07 | 0.69±0.04 | 0.082 |
|  | Uric acid (mg/dL) | 2.34±0.70 | 2.34±1.11 | 3.58±1.02 | 0.265 |
|  | Urea (mg/dL) | 61.32±4.93 | 64.6±12.24 | 56.93±6.98 | 0.579 |
| **Heart/muscle** | Creatine kinase (U/L) ^#^ | 222.25±14.53 | 466.17±597.33 | 456.29±174.59 | 0.301 |
| **Metabolism** | Triglycerides (mg/dL) | 80.88±5.80 | 70.05±20.41 | 46.30±11.77 | 0.057 |
|  | Cholesterol (mg/dL) | 96.05±25.34 | 67.85±13.2 | 80.75±15.95 | 0.264 |
|  | Glucose (mg/dL) | 231.98±32.03 | 236.62±28.36 | 222.70±36.22 | 0.869 |
| **Haematology parameters** | | | | | |
| **White Blood Cells** | White blood cell (WBC; 10^3^ cells/µL) | 2.48±0.28 | 2.94±0.73 | 2.33±0.08 | 0.297 |
|  | Neutrophil (10^3^ cells/µL) | 0.10±0.04 | 0.13±0.01 | 0.11±0.02 | 0.533 |
|  | Lymphocyte (10^3^ cells/µL) | 2.29±0.25 | 2.77±0.73 | 2.10±0.06 | 0.251 |
|  | Monocyte (10^3^ cells/µL) | 0.08±0.04 | 0.04±0.01 | 0.09±0.04 | 0.136 |
|  | Eosinophil (10^3^ cells/µL) | 0.01±0.02 | 0.01±0.01 | 0.01±0.01 | 0.927 |
|  | Basophil (10^3^ cells/µL) | 0.01±0.01 | 0.01±0.01 | 0.01±0.01 | 0.579 |
| **Red Blood Cells** | Hematocrit (%) | 40.93±1.25 | 40.73±4.13 | 41.97±1.89 | 0.841 |
|  | Red blood cell count (RBC; 10^6^ cells/µL) | 8.74±0.17 | 8.59±0.57 | 9.1±0.34 | 0.336 |
|  | Hemoglobin (g/dL) | 14.82±0.47 | 14.56±0.67 | 15.47±0.24 | 0.146 |
|  | Mean cell volume (MCV; fL) | 46.80±0.56 | 47.33±1.82 | 46.1±0.56 | 0.465 |
|  | Mean cell hemoglobin (MCH; pg) | 16.97±0.81 | 16.97±0.35 | 17.00±0.61 | 0.997 |
|  | Mean cell hemoglobin concentration (MCHC; g/dL) | 36.27±2.15 | 35.87±1.89 | 36.93±1.70 | 0.798 |
|  | Red blood cell distribution width (RDW; %) | 19.53±1.16 | 19.97±0.8 | 21.33±1.23 | 0.185 |
|  | Red Cell Distribution Width Standard Deviation (RDW-SD; fL) | 22.20±0.60 | 24.10±1.41 | 23.1±0.26 | 0.105 |
| **Platelets** | Platelet count (10^3^ cells/µL) ^#^ | 354.13±116.67 | 539.3±113.57 | 672.37±23.59 | 0.058 |
|  | Mean platelet volume (MPV; fL) | 6.39±0.87 | 5.93±0.22 | 6.23±0.26 | 0.595 |

* Non-significant difference (p > 0.05)

^#^ one-way ANOVA was used to compare the 3 groups, except for creatine kinase and platelet count (Kruskal-Wallis Test was used)

**Chemistry**

**General methods**. Chemical reagents and anhydrous solvents were purchased from Sigma-Aldrich and were used without further purification. Solvents for extraction and column chromatography were distilled prior to use. The 2-aminoazoles (compounds **2** and **5**) used in this manuscript were purchased from sigma-Aldrich. TLC analysis was performed with silica gel plates (0.25 mm, E. Merck, 60 F_254_) using iodine and a UV lamp for visualization. Melting points were measured with a Stuart Melting Point Apparatus (SMP30) in Celsius degrees and were uncorrected. ^1^H, ^13^C NMR and 2D-NMR experiments were performed on a 500 MHz instrument. Chemical shifts are reported in parts per million (ppm) downstream from the internal tetramethylsilane standard. Spin multiplicities are described as s (singlet), d (doublet), dd (double doublets), t (triplet), (td) triple doublets or m (multiplet). Coupling constants are reported in Hertz (Hz). ESI mass spectrometry was performed on a Q-TOF high-resolution mass spectrometer or Q-TOF Ultim LC-MS. Infrared spectra were recorded on a Perkin-Elmer Spectrum Two UATR FT-IR instrument and reported in unit of cm^-1^. Single-crystal X-ray diffraction data were collected using an Oxford Diffraction XCalibur, equipped with (Mo) X-ray Source (*λ* = 0.71073 Å) at 293(2) K.

**General Reaction Procedure for the Preparation of Compounds 1a-g.** Compounds **1a-g** were prepared according to a precedent procedure and the spectroscopic data as well as melting point ranges for the known compounds (**1a**, **1c** and **1g)** are matching that published in references 5a and 12.^5a,12^

***Methyl (E)-4-(2-formylphenoxy)but-2-enoate (1a).***

White solid (1.1g, 85% yield). mp 63-65 °C (lit. mp 63-65 °C)

IR (neat): 2953, 2871, 1715, 1682, 1302, 1238, 1017, 761 cm^-1^.

^1^H NMR (500 MHz, CDCl_3_) δ 10.56 (s, 1H), 7.90 – 7.86 (m, 1H), 7.63 – 7.50 (m, 1H), 7.20 – 7.06 (m, 2H), 6.96 (d, *J* = 8.4 Hz, 1H), 6.31 – 6.18 (m, 1H), 4.87 – 4.84 (m, 2H), 3.79 (s, 3H).

^13^C NMR (125 MHz, CDCl_3_) δ 189.4, 166.3, 160.2, 141.6, 136.0, 129.0, 125.2, 122.2, 121.6, 112.6, 66.9, 51.9.

HRMS (ESI-TOF) m/z: [M+Na]^+^ calcd for: C_12_H_12_O_4_Na; 243.0633 found: 243.0628.

***Methyl (E)-4-(2,4-difluoro-6-formylphenoxy)but-2-enoate (1b).***

White solid (1.03g, 81% yield). mp 122–124 °C.

IR (neat): 2958, 1736, 1690, 1377, 1325, 1222, 1121, 977, 689 cm^-1^.

^1^H NMR (400 MHz, CDCl_3_) δ 10.38 (s, 1H), 7.41 – 7.32 (m, 1H), 7.22 – 7.01 (m, 2H), 6.29 – 6.19 (m, 1H), 4.97 – 4.83 (m, 2H), 3.79 (s, 3H).

^13^C NMR (100 MHz, CDCl_3_) δ 187.1, 166.1, 157.8 (dd, ^1^*J_C-F_* = 248.0 Hz, ^2^*J_C-F_* = 12.6 Hz), 155.1 (dd, ^1^*J_C-F_* = 250.0 Hz, ^2^*J_C-F_* = 12.6 Hz), 145.2 (dd, ^2^*J_C-F_* = 15.1 Hz, ^3^*J_C-F_* = 5.0 Hz), 141.1, 130.4 (dd, ^2^*J_C-F_* = 8.8 Hz, ^3^*J_C-F_* = 2.5 Hz), 122.7, 111.1 (dd, ^2^*J_C-F_* = 34.0 Hz, ^2^*J_C-F_* = 27.7 Hz), 109.6 (dd, ^2^*J_C-F_* = 29.0 Hz, ^3^*J_C-F_* = 3.8 Hz), 73.2, 51.9.

HRMS (ESI-TOF) m/z: [M+Na]^+^ calcd for: C_12_H_10_F_2_O_4_Na; 279.0444 found: 279.0421.

***Methyl (E)-4-((1-formylnaphthalen-2-yl)oxy)but-2-enoate (1c)****.*

Off White solid (1.21g, 90% yield). mp 87–89 °C.

IR (neat): 2955, 1719, 1663, 1508, 1436, 1269, 1172, 821, 757 cm^-1^.

^1^H NMR (500 MHz, CDCl_3_) δ 10.99 (s, 1H), 9.29 (d, *J* = 8.7 Hz, 1H), 8.07 (d, *J* = 9.1 Hz, 1H), 7.80 (d, *J* = 8.0 Hz, 1H), 7.69 – 7.63 (m, 1H), 7.47 (t, *J* = 7.5 Hz, 1H), 7.26 – 7.08 (m, 2H), 6.27 (dd, *J* = 15.8, 1.7 Hz, 1H), 4.98 (s, 2H), 3.80 (s, 3H).

^13^C NMR (125 MHz, CDCl_3_) δ 191.6, 166.3, 162.3, 141.5, 137.7, 131.6, 130.2, 129.0, 128.4, 125.3, 125.2, 122.4, 117.4, 113.3, 67.9, 52.0.

HRMS (ESI-TOF) m/z: [M+Na]^+^ calcd for: C_16_H_14_O_4_Na; 293.0789 found: 293.0770.

***Methyl (E)-4-((2-formylnaphthalen-1-yl)oxy)but-2-enoate (1d)****.*

Yellowish solid (1.09g, 81% yield). mp 98–100 °C.

IR (neat): 2850, 1715, 1673, 1432, 1308, 1231, 1168, 1090, 913, 812, 744 cm^-1^.

^1^H NMR (500 MHz, CDCl_3_) δ 10.57 (s, 1H), 8.19 (d, *J* = 8.4 Hz, 1H), , 7.91 (dd, *J* = 10.9, 8.4 Hz, 2H), 7.79 – 7.57 (m, 3H), 7.21 (d, *J* = 15.7Hz, 1H), 6.49 (d, *J* = 15.7Hz, 1H), 4.89 (dd, *J* = 4.2, 2.0 Hz, 2H), 3.84 (s, 3H).

^13^C NMR (125 MHz, CDCl_3_) δ 189.2, 166.4, 160.4, 141.6, 138.1, 129.6, 128.5, 127.7, 127.2, 125.2, 125.1, 122.9, 122.8, 122.1, 75.9, 51.9.

HRMS (ESI-TOF) m/z: [M+Na]^+^ calcd for: C_16_H_14_O_4_Na; 293.0789 found: 293.0765.

***Methyl (E)-4-((3-formylnaphthalen-2-yl)oxy)but-2-enoate (1e)****.*

White solid (0.97g, 72% yield). mp 94–96 °C.

IR (neat): 2954, 1720, 1664, 1431, 1306, 1175, 1022, 823, 759 cm^-1^.

^1^H NMR (500 MHz, Acetone-d_6_) δ 10.66 (s, 1H), 8.40 (s, 1H), 8.05 (dd, *J* = 8.2, 0.6 Hz, 1H), 7.89 (dd, *J* = 8.2, 0.6 Hz, 1H), 7.65 – 7.59 (m, 1H), 7.51 – 7.41 (m, 1H), 7.23 (d, *J* = 15.8 Hz, 1H), 6.37 (d, *J* = 15.8 Hz, 1H, 5.10 (dd, *J* = 4.1, 2.1 Hz, 2H), 3.74 (s, 3H).

^13^C NMR (125 MHz, Acetone-d_6_) δ 188.9, 165.8, 156.0, 142.4, 137.5, 130.7, 129.7, 129.2, 128.0, 126.8, 126.0, 124.9, 121.3, 108.0, 66.9, 50.9.

HRMS (ESI-TOF) m/z: [M+Na]^+^ calcd for: C_16_H_14_O_4_Na; 293.0789 found: 293.0765.

***Methyl (E)-4-(5-(diethylamino)-2-formylphenoxy)but-2-enoate (1f)****.*

Yellowish solid (0.8g, 55% yield). mp 114–116 °C.

IR (neat): 2973, 2843, 1721, 1587, 1354, 1266, 1114, 808, 693 cm^-1^.

^1^H NMR (500 MHz, CD_3_OD) δ 10.04 (s, 1H), 7.66 (d, *J* = 9.0 Hz, 1H), 7.20 – 7.12 (m, 1H), 6.43 (dd, *J* = 9.0, 2.1 Hz, 1H), 6.32 – 6.21 (m, 1H), 6.13 (dd, *J* = 18.8, 1.9 Hz, 1H), 4.94 – 4.90 (m, 2H), 3.77 (s, 3H), 3.50 (q, *J* = 7.1 Hz, 4H), 1.22 (t, *J* = 7.1 Hz, 6H).

^13^C NMR (125 MHz, CD_3_OD) δ 186.9, 166.6, 163.0, 154.5, 143.0, 130.5, 120.9, 113.5, 104.8, 93.5, 66.4, 50.8, 44.4, 11.4.

HRMS (ESI-TOF) m/z: [M+Na]^+^ calcd for: C_16_H_21_NO_4_Na; 314.1368 found: 314.1345.

***Methyl (E)-4-((9-formyl-2,3,6,7-tetrahydro-1H,5H-pyrido[3,2,1-ij]quinolin-8-yl)oxy)but-2-enoate (1g)****^5a^****.***

Yellowish solid (1.57g, 74% yield). mp 78–80 °C.

IR (neat): 2941, 2838, 1724, 1650, 1587, 1312, 1162, 978, 824, 721 cm^-1^.

^1^H NMR (500 MHz, CDCl_3_) δ 9.94 (s, 1H), 7.33 (s, 1H), 7.13 – 7.07 (m, 1H), 6.35 – 6.29 (m, 1H), 4.58 (dd, *J* = 4.2, 2.1 Hz, 2H), 3.79 (s, 3H), 3.39 – 3.24 (m, 4H), 2.76 – 2.70 (m, 4H), 2.10 – 1.87 (m, 4H).

^13^C NMR (125 MHz, CDCl_3_) δ 187.3, 166.7, 158.7, 149.1, 142.9, 127.8, 121.4, 117.6, 116.9, 112.5, 73.4, 51.8, 50.2, 49.8, 27.4, 21.4, 21.1, 20.8.

HRMS (ESI-TOF) m/z: [M+Na]^+^ calcd for: C_18_H_21_NO_4_Na; 338.1368 found: 338.1345.

**General Reaction Procedure for the Preparation of Compounds 4a-b/6a-y**. Ytterbium triflate (20 mol%) and sodium sulfate (1.0 mmol) were added to a solution of aldehyde (**1**, 0.5 mmol) and 2-aminoazine (**2/5,** 0.5 mmol) in MeOH:DCM (1.5:0.5 mL) at rt. After 45 mins, tert-Butyl isocyanide (0.55 mmol) was introduced and stirring was continued for 12-15h. After completion of step-1, an additional 30 mol% of ytterbium triflate was added and the reaction was continued at 70 °C for 3-12h. After completion, MeOH and DCM were removed and the crude was purified on flash column chromatography, using a gradient of EtOAc/hexane or MeOH/DCM as eluents to deliver the pure products **4a-b**/**6a-y**.

***14-(Tert-butylamino)-7-(2-methoxy-2-oxoethyl)-6,7-dihydrobenzo[f]pyrazino[2',1':2,3] imidazo[1,5-d][1,4]oxazepin-8-ium trifluoromethanesulfonate (4a).***

Yellowish solid (137 mg, 52%). mp 80–82 °C.

IR (neat): 3301, 2970, 1731, 1486, 1440, 1248, 1154, 1028 cm^-1^.

^1^H NMR (500 MHz, CD_3_OD) δ 9.74 (s, 1H), 8.87 (dd, *J* = 4.5, 1.1 Hz, 1H), 8.56 (d, *J* = 4.5, 1.1 Hz, 1H), 8.40 (d, *J* = 8.0, 1H), 7.61 – 7.55 (m, 1H), 7.31 – 7.23 (m, 2H), 6.04 – 5.95 (m, 1H), 5.00 (bs, 0.7H), 4.82 (dd, *J* = 13.1, 3.7 Hz, 1H), 4.71 (d, *J* = 13.1 Hz, 1H), 3.60 (s, 3H), 3.10 (dd, *J* = 18.1, 3.6 Hz, 1H), 2.79 (dd, *J* = 18.1, 10.2 Hz, 1H, 1.12 (s, 9H).

^13^C NMR (125 MHz, CD_3_OD) δ 170.5, 155.0, 137.9, 133.9, 133.1, 1317, 131.2, 130.0, 127.6, 122.4, 120.8, 120.4 (q, *^1^J_C-F_* = 318.8 Hz), 117.5, 113.5, 72.5, 56.8, 55.4, 51.3, 33.3, 28.9.

HRMS (ESI-TOF) m/z: [M-CF_3_SO_3_]^+^ calcd for: C_21_H_25_N_4_O_3_; 381.1926 found: 381.1962.

***16-(Tert-butylamino)-7-(2-methoxy-2-oxoethyl)-6,7-dihydrobenzo[6',7'][1,4]oxazepino [4',5':3,4]imidazo[1,2-a]quinolin-8-ium trifluoromethanesulfonate (4b).***

White solid (133 mg, 46%). mp 85–87 °C.

IR (neat): 3335, 2969, 1737, 1508, 1461, 1251, 1154, 1028 cm^-1^.

^1^H NMR (500 MHz, CD_3_OD) δ 10.14 (d, *J* = 8.8 Hz, 1H), 8.44 (d, *J* = 9.5 Hz, 1H), 8.21 (d, *J* = 9.3 Hz, 2H), 8.07 (d, *J* = 7.6 Hz, 1H), 7.99 (t, *J* = 7.7 Hz, 1H), 7.84 (t, *J* = 7.4 Hz, 1H), 7.61 (t, *J* = 7.2 Hz, 1H), 7.37 (t, *J* = 7.4 Hz, 1H), 7.31 (d, *J* = 8.1 Hz, 1H), 5.96 – 5.84 (m, 1H), 5.16 (s, 1H), 4.86 – 4.83 (m, 1H), 4.71 (dd, *J* = 12.7, 2.2 Hz, 1H), 3.55 (s, 3H), 2.98 (dd, *J* = 17.7, 3.8 Hz, 1H), 2.71 (dd, *J* = 17.7, 10.3 Hz, 1H), 1.01 (s, 9H).

^13^C NMR (125 MHz, CD_3_OD) δ 170.3, 155.0, 136.9, 135.4, 133.6, 132.7, 131.6, 131.4, 130.4, 129.8, 129.4, 127.6, 125.1, 123.7, 121.1, 120.1 (q, *^1^J_C-F_* = 320.0 Hz), 119.2, 116.9, 108.8, 73.9, 56.8, 54.2, 51.2, 34.9, 28.4.

HRMS (ESI-TOF) m/z: [M-CF_3_SO_3_]^+^ calcd for: C_26_H_28_N_3_O_3_; 430.2130 found: 430.2156.

***15-(Tert-butylamino)-7-(2-methoxy-2-oxoethyl)-6,7-dihydrobenzo[f]benzo[4',5']thiazolo [2',3':2,3]imidazo[1,5-d][1,4]oxazepin-8-ium trifluoromethanesulfonate (6a).***

White solid (152 mg, 52%). mp 122-124 ^o^C.

IR (neat): 3334, 1737, 1518, 1445, 1251, 1161, 1029 cm^-1^.

^1^H NMR (500 MHz, CD_3_OD) δ 8.75 (d, *J* = 8.3 Hz, 1H), 8.19 (d, *J* = 8.1 Hz, 1H), 8.07 (dd, *J* = 7.8, 1.4 Hz, 1H), 7.79 (t, *J* = 7.9 Hz, 1H), 7.71 (t, *J* = 7.8 Hz, 1H), 7.55 (t, *J* = 7.8 Hz, 1H), 7.31 (t, *J* = 7.6 Hz, 1H), 7.26 (d, *J* = 8.2 Hz, 1H) 5.34 – 5.27 (m, 1H), 5.07 (bs, 0.5H), 4.79 (dd, *J* = 12.7, 3.2 Hz, 1H), 4.67 (dd, *J* = 12.7, 4.8 Hz, 1H), 3.66 (s, 3H), 3.08 (dd, *J* = 18.0, 3.7 Hz, 1H), 2.90 (dd, *J* = 18.0, 10.2 Hz, 1H), 1.11 (s, 9H).

^13^C NMR (125 MHz, CD_3_OD) δ 170.3, 154.3, 132.4, 132.3, 131.0, 130.9 (2C), 130.7, 129.5, 127.5, 127.4, 124.7, 123.4, 121.7, 121.1, 120.4 (q, *^1^J_C-F_* = 318.8 Hz), 119.1, 117.0, 116.9, 73.1, 58.3, 56.4, 51.5, 33.5, 28.8.

HRMS (ESI-TOF) m/z: [M-CF_3_SO_3_]^+^ calcd for: C_24_H_26_N_3_O_3_S; 436.1694 found: 436.1701.

***13-(Tert-butylamino)-7-(2-methoxy-2-oxoethyl)-6,7-dihydrobenzo[f]thiazolo[2',3':2,3] imidazo[1,5-d][1,4]oxazepin-8-ium trifluoromethanesulfonate (6b).***

White solid (120 mg, 45%). mp 97-99 ^o^C.

IR (neat): 3300, 2966, 1731, 1487, 1440, 1250, 1154, 1028 cm^-1^.

^1^H NMR (500 MHz, CD_3_OD) δ 8.25 (d, *J* = 8.0 Hz, 1H), 8.15 (d, *J* = 4.3 Hz, 1H), 7.70 (d, *J* = 4.3 Hz, 1H), 7.51 – 7.46 (m, 1H), 7.28 – 7.17 (m, 2H), 5.29 – 5.23 (m, 1H), 4.79 (bs, 0.5H), 4.68 – 4.64 (m, 2H), 3.67 (s, 3H), 3.09 (dd, *J* = 18.0, 3.6 Hz, 1H), 2.89 (dd, *J* = 18.0, 10.3 Hz, 1H), 1.10 (s, 9H).

^13^C NMR (125 MHz, CD_3_OD) δ 170.6, 154.2, 145.1, 131.8, 130.6, 129.4, 128.1, 122.7, 120.8, 120.2 (q, *^1^J_C-F_* = 318.8 Hz), 120.1, 117.7, 115.9, 72.4, 58.4, 56.0, 51.5, 33.3, 28.7.

HRMS (ESI-TOF) m/z: [M-CF_3_SO_3_]^+^ calcd for: C_20_H_24_N_3_O_3_S; 386.1538 found: 386.1547.

***13-(Tert-butylamino)-7-(2-methoxy-2-oxoethyl)-10-methyl-11-phenyl-6,7-dihydrobenzo[f] thiazolo[2',3':2,3]imidazo[1,5-d][1,4]oxazepin-8-ium trifluoromethanesulfonate (6c).***

White solid (171 mg, 55%). mp 105-107 ^o^C.

IR (neat): 3339, 1732, 1489, 1440, 1255, 1150, 1029 cm^-1^.

^1^H NMR (500 MHz, CD_3_OD) δ 8.06 (dd, *J* = 7.8, 1.3 Hz, 1H), 7.69 – 7.62 (m, 5H), 7.52 – 7.44 (m, 1H), 7.25 – 7.14 (m, 2H), 5.29 – 5.22 (m, 1H), 4.73 – 4.63 (m, 2H), 3.70 (s, 3H), 3.58 (s, 0.8H), 3.10 (dd, *J* = 18.1, 3.5 Hz, 1H), 2.89 (dd, *J* = 18.1, 10.4 Hz, 1H), 2.49 (s, 3H), 0.53 (s, 9H).

^13^C NMR (125 MHz, CD_3_OD) δ 172.0, 155.7, 144.9, 133.3, 132.8, 132.5, 132.4, 131.5, 131.1, 131.0, 130.0, 128.8, 128.5, 124.2, 122.0, 121.8 (q, *^1^J_C-F_* = 318.8 Hz), 117.3, 74.1, 59.9, 56.7, 52.9, 34.8, 29.6, 13.0.

HRMS (ESI-TOF) m/z: [M-CF_3_SO_3_]^+^ calcd for: C_27_H_30_N_3_O_3_S; 476.2007 found: 476.2019.

***13-(Tert-butylamino)-2,4-difluoro-7-(2-methoxy-2-oxoethyl)-10-methyl-11-phenyl-6,7-dihydrobenzo [f]thiazolo[2',3':2,3]imidazo[1,5-d][1,4]oxazepin-8-ium trifluoromethanesulfonate (6d).***

White solid (148 mg, 45%). mp 110-112 ^o^C.

IR (neat): 2968, 1738, 1480, 1441, 1256, 1151, 1029 cm^-1^

^1^H NMR (500 MHz, CD_3_OD) δ 7.75 (d, *J* = 9.3 Hz, 1H), 7.70 – 7.63 (m, 5H), 7.31 – 7.23 (m, 1H), 5.34 – 5.27 (m, 1H), 4.80 – 4.69 (m, 2H), 3.71 (s, 3H), 3.63 (s, 1H), 3.14 (dd, *J* = 18.0, 3.7 Hz, 1H), 2.97 (dd, *J* = 18.0, 10.1 Hz, 1H), 2.49 (s, 3H), 0.57 (s, 9H).

^13^C NMR (125 MHz, CD_3_OD) δ 170.4, 157.2 (dd, *^1^J_C-F_* = 244.0 Hz, *^2^J_C-F_* = 12.6 Hz), 154.3 (dd, *^1^J_C-F_* = 252.0 Hz, *J_C-F_* = *^2^*12.6 Hz), 144.1, 139.2 (dd, *^3^J_C-F_* = 8.8 Hz, ^3^*J_C-F_* = 3.8 Hz), 131.1, 130.9, 130.3, 130.2, 128.7, 127.9, 127.6, 126.9, 120.4 (q, *^1^J_C-F_* = 318.8 Hz), 119.73 (d, *^2^J_C-F_* = 10.8 Hz). 112.0 (dd, *^2^J_C-F_* = 26.5 Hz, *^3^J_C-F_* = 5.0 Hz), 106.5 (dd, *^2^J_C-F_* = 27.7 Hz, *^2^J_C-F_* = 23.9 Hz,), 73.4, 57.9, 55.7, 51.2, 33.5, 28.3, 11.6.

HRMS (ESI-TOF) m/z: [M-CF_3_SO_3_]^+^ calcd for: C_27_H_28_F_2_N_3_O_3_S; 512.1819 found: 512.1819.

***15-(Tert-butylamino)-9-(2-methoxy-2-oxoethyl)-12-methyl-13-phenyl-8,9-dihydronaphtho [1,2-f]thiazolo[2',3':2,3]imidazo[1,5-d][1,4]oxazepin-10-ium trifluoromethanesulfonate (6e).***

White solid (162 mg, 48%); Mixture of diastereoisomers (64:36).

IR (neat): 3372, 1725, 1514, 1437, 1250, 1150, 1727 cm^-1^.

^1^H NMR (500 MHz, CDCl_3_) δ 8.06 (d, *J* = 8.4 Hz, 0.36H), 8.01 (d, *J* = 8.8 Hz, 0.36H), 7.97 (d, *J* = 8.8 Hz, 0.64H), 7.93 – 7.86 (m, 1.8H), 7.71 – 7.50 (m, 6H), 7.36 –7.29 (m, 1H), 5.46 – 5.36 (m, 0.64H), 5.14 – 5.05 (dd, *J* = 12.4, 6.2 Hz, 0.64H), 5.05 – 4.97 (m, 0.36H), 4.66 – 4.56 (m, 0.64H), 4.46 (d, *J* = 12.4 Hz, 0.64H), 3.84 (dd, *J* = 16.7, 5.8 Hz, 0.36H), 3.71 (s, 3H), 3.57 (s, 2H), 3.17 (dd, *J* = 16.7, 8.5 Hz, 0.36H), 2.70 – 2.48 (m, 2.2H), 2.42 (two s, 3H), 0.18 (two s, 9H).

^13^C NMR (125 MHz, CDCl_3_) δ 170.6, 168.8, 154.5, 152.7, 144.2, 139.8, 133.67, 133.62, 131.5, 131.4, 131.3, 131.2, 130.9, 130.85, 130.82, 130.80, 130.7, 130.5, 130.2, 129.8, 129.7, 129.4, 128.9, 128.7, 128.5, 128.46, 128.41, 127.3, 126.9, 126.8, 126.3, 126.2, 126.0, 125.9, 125.5, 124.7, 120.9 (q, *^1^J_C-F_* = 321.3 Hz) , 120.7, 119.6, 117.1, 115.2, 76.6, 75.7, 56.4, 56.0, 55.9, 53.6, 52.8, 52.4, 37.3, 31.8, 29.04, 29.01, 13.2, 13.0.

HRMS (ESI-TOF) m/z: [M-CF_3_SO_3_]^+^ calcd for: C_31_H_32_N_3_O_3_S; 526.2164 found: 526.2175.

***13-(4-bromophenyl)-15-(tert-butylamino)-9-(2-methoxy-2-oxoethyl)-12-methyl-8,9-dihydronaphtho[1,2-f]thiazolo[2',3':2,3]imidazo[1,5-d][1,4]oxazepin-10-ium trifluoromethanesulfonate (6f).***

White solid (195 mg, 52%); Mixture of diastereoisomers (64:36).

IR (neat): 2966, 1723, 1471, 1431, 1254, 1152, 1031 cm^-1^.

^1^H NMR (500 MHz, CDCl_3_) δ 8.25 – 8.14 (m, 1.36H), 8.09 – 7.96 (m, 2.36H), 7.88 – 7.57 (m, 7.36H), 7.54 – 7.44 (m, 1.36), 5.43 – 5.35 (m, 0.66H), 5.10 – 5.01 (m, 1.38H), 4.70 – 4.49 (m, 1.6H), 3.79 – 3.72 (m, 1.6H), 3.64 (s, 0.56H), 3.54 (s, 2H), 3.01 – 2.89 (m, 1H), 2.79 – 2.69 (m, 0.63H), 2.55 – 2.47 (two s, 3H), 2.44 – 2.35 (m, 0.6H), 0.31 – 0.21 (two s, 9H).

^13^C NMR (125 MHz, CDCl_3_) δ 171.6, 170.5, 155.9, 154.2, 145.7, 144.8, 144.0, 141.5, 134.9, 134.8, 134.5, 133.2, 133.1, 132.9, 132.7, 132.4, 131.8, 131.6, 131.5, 131.4, 131.3, 131.25, 131.20, 129.9, 129.8, 129.5, 129.4, 129.0, 128.5, 128.4, 127.4, 127.26, 127.20, 126.3, 126.2, 125.7, 122.4, 121.9,121.8 (q, *^1^J_C-F_* = 319.4 Hz), 118.0, 116.9, 77.4, 76.5, 57.5, 56.47, 56.44, 55.2, 53.2, 52.6, 38.4, 32.0, 29.36, 29.33, 13.0, 12.8.

HRMS (ESI-TOF) m/z: [M-CF_3_SO_3_]^+^ calcd for: C_31_H_31_BrN_3_O_3_S; 604.1269 found: 604.1269.

***13-(Tert-butylamino)-7-(2-methoxy-2-oxoethyl)-10-methyl-11-phenyl-6,7-dihydronaphtho[2,1- f]thiazolo[2',3':2,3]imidazo[1,5-d][1,4]oxazepin-8-ium trifluoromethanesulfonate (6g).***

Yellowish solid (172 mg, 51%). mp 122-124 ^o^C.

IR (neat): 3364, 1736, 1441, 1415, 1257, 1149, 1031 cm^-1^.

^1^H NMR (500 MHz, CDCl_3_) δ 8.30 (dd, *J* = 8.2, 0.8 Hz, 1H), 8.09 (d, *J* = 8.9 Hz, 1H), 7.79 – 7.76 (m, 1H), 7.68 – 7.51 (m, 7H), 7.47 (d, *J* = 8.9 Hz, 1H), 5.47 – 5.40 (m, 1H), 5.03 – 4.94 (m, 2H), 3.69 (s, 3H), 3.06 – 2.91 (m, 2H), 2.43 (s, 3H), 0.56 (s, 9H).

^13^C NMR (125 MHz, CDCl_3_) δ 171.2, 150.0, 144.7, 135.0, 132.1, 130.8, 130.0, 129.8, 129.5, 128.3, 128.2, 128.0, 127.5, 127.3, 127.3, 126.7, 126.3, 123.2, 121.9, 120.9 (q, *^1^J_C-F_* = 318.8 Hz), 108.4, 72.9, 59.8, 57.3, 52.6, 33.5, 29.6, 13.2.

HRMS (ESI-TOF) m/z: [M-CF_3_SO_3_]^+^ calcd for: C_31_H_32_N_3_O_3_S; 526.2164 found: 526.2147.

***14-(Tert-butylamino)-5-(2-methoxy-2-oxoethyl)-2-methyl-1-phenyl-5,6-dihydronaphtho[2,3-f]thiazolo[2',3':2,3]imidazo[1,5-d][1,4]oxazepin-4-ium trifluoromethanesulfonate (6h).***

White solid (185 mg, 55%). mp 108-110 ^o^C.

IR (neat): 3315, 1733, 1496, 1443, 1258, 1160, 1029 cm^-1^.

^1^H NMR (500 MHz, CDCl_3_) δ 8.60 (s, 1H), 7.97 (d, *J* = 8.1 Hz, 1H), 7.79 (d, *J*= 8.1 Hz, 1H), 7.70 – 7.45 (m, 7H), 7.28 (s, 1H), 5.30 – 5.21 (m, 1H), 4.95 – 4.86 (m, 1H), 4.58 – 4.50 (m, 1H), 3.67 (s, 3H), 3.14 (s, 0.9H), 3.02 (dd, *J* = 17.7, 8.3 Hz, 1H), 2.81 (dd, *J* = 17.7, 3.9 Hz, 1H), 2.45 (s, 3H), 0.47 (s, 9H).

^13^C NMR (125 MHz, CDCl_3_) δ 170.4, 151.4, 142.9, 135.1, 132.2, 131.5, 131.4, 130.7, 130.4, 130.2, 129.5, 129.2, 128.8, 128.3, 127.1, 126.9, 126.8, 126.2, 120.9 (q, *^1^J_C-F_* = 320.0 Hz), 119.2, 117.9, 74.8, 56.8, 56.1, 52.7, 35.9, 29.6, 13.3.

HRMS (ESI-TOF) m/z: [M-CF_3_SO_3_]^+^ calcd for: C_31_H_32_N_3_O_3_S; 526.2164 found: 526.2172.

***11-(4-Bromophenyl)-13-(tert-butylamino)-3-(diethylamino)-7-(2-methoxy-2-oxoethyl)-10-methyl-6,7-dihydrobenzo[f]thiazolo[2',3':2,3]imidazo[1,5-d][1,4]oxazepin-8-ium trifluoromethanesulfonate (6i).*** White solid (185 mg, 48%). mp 176-178 ^o^C.

IR (neat): 2973, 1725, 1616, 1440, 1402, 1245, 1166, 1029 cm^-1^.

^1^H NMR (500 MHz, CD_3_OD) δ 7.79 (d, *J* = 8.4 Hz, 2H), 7.74 (d, *J* = 9.0 Hz, 1H), 7.56 (d, *J* = 8.4 Hz, 2H), 6.55 (dd, *J* = 9.0, 2.3 Hz, 1H), 6.38 (d, *J* = 2.3 Hz, 1H), 5.21 – 5.14 (m, 1H), 4.65 – 4.55 (m, 2H), 3.70 (s, 3H), 3.44 (q, *J* = 7.0 Hz, 4H), 3.09 (dd, *J* = 18.0, 3.3 Hz, 1H), 2.95 (dd, *J*= 18.0, 10.3 Hz, 1H), 2.48 (s, 3H), 1.21 (t, *J* = 7.0 Hz, 6H), 0.59 (s, 9H).

^13^C NMR (125 MHz, CD_3_OD) δ 170.8, 155.8, 150.7, 142.2, 133.0, 132.1, 132.0, 131.4, 130.1, 127.6, 127.3, 126.3, 123.9, 120.4 (q, *^1^J_C-F_* = 318.8 Hz), 106.6, 101.1, 100.9, 72.1, 58.6, 54.8, 51.4, 43.9, 33.0, 28.4, 11.6, 11.4.

HRMS (ESI-TOF) m/z: [M-CF_3_SO_3_]^+^ calcd for: C_31_H_38_BrN_4_O_3_S; 625.1847 found: 625.1837.

***13-(Tert-butylamino)-3-(diethylamino)-7-(2-methoxy-2-oxoethyl)-10,11-bis(4-methoxyphenyl)-6,7-dihydrobenzo[f]thiazolo[2',3':2,3]imidazo[1,5-d][1,4]oxazepin-8-ium trifluoromethanesulfonate (6j).*** Brownish solid (184 mg, 44%). mp 111-113 ^o^C.

IR (neat): 2968, 1732, 1611, 1515, 1497, 1247, 1153, 1031 cm^-1^.

^1^H NMR (500 MHz, CD_3_OD) δ 7.84 (d, *J* = 9.0 Hz, 1H), 7.49 – 7.40 (m, 2H), 7.27 – 7.22 (m, 2H), 7.07 (d, *J* = 8.1 Hz, 2H), 6.96 – 6.92 (m, 2H), 6.55 (dd, *J* = 9.0, 2.6 Hz, 1H), 6.38 (d, *J* = 2.5 Hz, 1H), 5.25 – 5.18 (m, 1H), 4.66 – 4.59 (m, 2H), 3.87 (s, 3H), 3.82 (s, 3H), 3.69 (s, 3H), 3.45 (q, *J* = 7.0 Hz, 4H), 3.11 (dd, *J* = 18.1, 3.4 Hz, 1H), 2.98 (dd, *J* = 18.1, 10.3 Hz, 1H), 1.21 (t, *J* = 7.0 Hz, 6H), 0.61 (s, 9H).

^13^C NMR (125 MHz, CD_3_OD) δ 172.3, 162.7, 162.3, 157.1, 152.0, 143.7, 134.3, 133.6, 132.4, 132.2, 131.4, 130.8, 129.4, 122.4, 121.8 (q, *^1^J_C-F_* = 318.8 Hz), 120.6, 115.6, 115.4, 108.0, 102.4, 102.1, 73.3, 60.1, 56.5, 56.0, 55.9, 52.9, 45.3, 34.4, 29.9, 12.8.

HRMS (ESI-TOF) m/z: [M-CF_3_SO_3_]^+^ calcd for: C_38_H_45_N_4_O_5_S; 669.3110 found: 669.3090.

***12-(Tert-butylamino)-10-(3,4-difluorophenyl)-6-(2-methoxy-2-oxoethyl)-2,3,5,6,15,16-hexahydro-1H,14H-pyrido[3,2,1-ij]thiazolo[2'',3'':2',3']imidazo[1',5':4,5][1,4]oxazepino [7,6-f]quinolin-7-ium trifluoromethanesulfonate (6k).***

White solid (210 mg, 54%). mp 158-160 ^o^C.

IR (neat): 3319, 2966, 1745, 1506, 1436, 1276, 1251, 1029 cm^-1^.

^1^H NMR (500 MHz, CD_3_OD) δ 7.81 – 7.74 (m, 1H), 7.63 – 7.54 (m, 2H), 7.51 – 7.44 (m, 1H), 7.28 (s, 1H), 5.22 – 5.14 (m, 1H), 4.66 (dd, *J* = 12.3, 4.5 Hz, 1H), 4.48 (dd, *J* = 12.3, 4.6 Hz, 1H), 4.19 (s, 0.8H), 3.66 (s, 3H), 3.31 – 3.22 (m, 4H), 3.08 – 2.88 (m, 2H), 2.85 – 2.71 (m, 4H), 2.06 – 1.92 (m, 4H), 0.61 (s, 9H).

^13^C NMR (125 MHz, CD_3_OD) δ 171.8, 152.9(dd, *^1^J_C-F_* = 243 Hz, *^2^J_C-F_* = 11.3 Hz), 151.8, 150.9 (dd, *^1^J_C-F_* = 249.0 Hz, *^2^J_C-F_* = 11.3 Hz), 147.2, 145.7, 135.7, 135.0, 129.2, 128.7 (2C) (d, *^2^J_C-F_* = 11.3 Hz), 126.6 (dd, *^3^J_C-F_* = 6.3 Hz, *^3^J_C-F_* = 3.8 Hz), 121.8 (q, *^1^J_C-F_* = 318.8 Hz), 121.5 (d, *^2^J_C-F_* = 11.3 Hz), 118.7 (d, *^2^J_C-F_* = 17.6 Hz), 118.3, 116.5, 113.6, 105.5, 74.1, 58.4, 56.3, 52.8, 50.8, 50.3, 35.5, 29.8, 28.3, 22.8, 22.2, 22.1.

HRMS (ESI-TOF) m/z: [M-CF_3_SO_3_]^+^ calcd for: C_32_H_35_F_2_N_4_O_3_S; 593.2397 found: 593.2417.

***10-(4-Bromophenyl)-12-(tert-butylamino)-6-(2-methoxy-2-oxoethyl)-2,3,5,6,15,16-hexahydro-1H,14H-pyrido[3,2,1-ij]thiazolo[2'',3'':2',3']imidazo[1',5':4,5][1,4]oxazepino [7,6-f]quinolin-7-ium trifluoromethanesulfonate (6l).***

White solid, (199 mg, 51%). mp 178-180 ^o^C.

IR (neat): 3329, 1735, 1504, 1376, 1275, 1251, 1161, 1029 cm^-1^.

^1^H NMR (500 MHz, CD_3_OD) δ 7.74 (d, *J* = 8.3 Hz, 2H), 7.66 (d, *J* = 8.3 Hz, 2H), 7.56 (s, 1H), 7.29 (s, 1H), 5.22 – 5.14 (m, 1H), 4.65 (dd, *J* = 12.3, 4.4 Hz, 1H), 4.49 (dd, *J* = 12.3, 4.6 Hz, 1H), 3.66 (s, 3H), 3.31 – 3.22 (m, 4H), 3.04 – 2.91 (m, 2H), 2.84 – 2.72 (m, 4H), 2.02 – 1.94 (m, 4H), 0.58 (s, 9H).

^13^C NMR (125 MHz, CD_3_OD) δ 170.5, 150.4, 145.8, 144.4, 135.4, 133.5, 132.1, 131.1, 127.7, 127.3, 127.2, 124.0, 120.4 (q, *^1^J_C-F_* = 318.8 Hz), 117.3, 114.5, 112.1, 104.1, 72.7, 57.0, 54.9, 51.4, 49.4, 48.9, 34.1, 28.4, 26.9, 21.4, 20.8, 20.7. HRMS (ESI-TOF) m/z: [M-CF_3_SO_3_]^+^ calcd for: C_32_H_36_BrN_4_O_3_S; 635.1691 found: 635.1710.

***12-(Tert-butylamino)-10-(2,5-dimethoxyphenyl)-6-(2-methoxy-2-oxoethyl)-2,3,5,6,15,16-hexahydro-1H,14H-pyrido[3,2,1-ij]thiazolo[2'',3'':2',3']imidazo[1',5':4,5][1,4]oxazepino [7,6-f]quinolin-7-ium trifluoromethanesulfonate (6m).***

White solid (183 mg, 48%). mp 160-162 ^o^C.

IR (neat): 2955, 1744, 1606, 1488, 1463, 1280, 1225, 1136, 1032 cm^-1^.

^1^H NMR (500 MHz, CD_3_OD) δ 7.47 (s, 1H), 7.35 (s, 1H), 7.22 – 7.14 (m, 3H), 5.20 – 5.12 (m, 1H), 4.62 (dd, *J* = 12.3, 4.0 Hz, 1H), 4.50 (dd, *J* = 12.3, 4.8 Hz, 1H), 3.86 (s, 3H), 3.84 (s, 3H), 3.68 (s, 3H), 3.40 (s, 0.3H), 3.29 – 3.19 (m, 4H), 3.09 – 2.90 (m, 2H), 2.83 – 2.72 (m, 4H), 2.05 – 1.93 (m, 4H), 0.60 (s, 9H).

^13^C NMR (125 MHz, CD_3_OD) δ 170.5, 154.0, 151.8, 150.4, 145.6, 143.7, 132.6, 131.7, 127.7, 127.5, 120.4 (q, *^1^J_C-F_* = 318.8 Hz), 117.9, 117.8, 117.0, 116.8, 114.8, 112.2, 111.7, 103.8, 72.4, 57.2, 55.5, 55.1, 54.9, 51.4, 49.4, 48.9, 34.0, 28.5, 26.9, 21.5, 20.8, 20.8.

HRMS (ESI-TOF) m/z: [M-CF_3_SO_3_]^+^ calcd for: C_34_H_41_N_4_O_5_S; 617.2797 found: 617.2758.

***10-(4-Acetamidophenyl)-12-(tert-butylamino)-6-(2-methoxy-2-oxoethyl)-2,3,5,6,15,16-hexahydro-1H,14H-pyrido[3,2,1-ij]thiazolo[2'',3'':2',3']imidazo[1',5':4,5][1,4]oxazepino [7,6-f]quinolin-7-ium trifluoromethanesulfonate (6n).***

Yellowish solid (171 mg, 45%). mp 178-180 ^o^C.

IR (neat): 2944, 1733, 1689, 1507, 1438, 1276, 1253, 1172, 1032 cm^-1^.

^1^H NMR (500 MHz, CD_3_OD) δ 7.79 (d, *J* = 8.6 Hz, 2H), 7.67 (d, *J* = 8.6 Hz, 2H), 7.45 (s, 1H), 7.32 (s, 1H), 5.22 – 5.13 (m, 1H), 4.64 (dd, *J* = 12.3, 4.4 Hz, 1H), 4.49 (dd, *J* = 12.3, 4.7 Hz, 1H), 3.94 (s, 0.3H), 3.66 (s, 3H), 3.29 – 3.21 (m, 4H), 3.05 – 2.90 (m, 2H), 2.84 – 2.72 (m, 4H), 2.19 (s, 3H), 2.05 – 1.94 (m, 4H), 0.58 (s, 9H).

^13^C NMR (125 MHz, CD_3_OD) δ 170.5, 170.4, 150.4, 145.7, 144.3, 140.5, 136.3, 133.0, 130.8, 127.8, 127.4, 123.2, 120.4 (q, *^1^J_C-F_* = 318.8 Hz), 118.8, 117.2, 113.3, 112.0, 104.1, 72.6, 57.0, 55.0, 51.4, 49.4, 48.9, 34.1, 28.4, 26.9, 22.6, 21.5, 20.8, 20.7.

HRMS (ESI-TOF) m/z: [M-CF_3_SO_3_]^+^ calcd for: C_34_H_40_N_5_O_4_S; 614.2801 found: 614.2795.

***12-(Tert-butylamino)-10-(2,3-dihydrobenzo[b][1,4]dioxin-5-yl)-6-(2-methoxy-2-oxoethyl)-2,3,5,6,15,16-hexahydro-1H,14H-pyrido[3,2,1-ij]thiazolo[2'',3'':2',3']imidazo[1',5':4,5] [1,4]oxazepino[7,6-f]quinolin-7-ium trifluoromethanesulfonate (6o).***

Greenish solid (183 mg, 48%). mp 184-186 ^o^C.

IR (neat): 2939, 1732, 1610, 1496, 1436, 1252, 1154, 1028 cm^-1^.

^1^H NMR (500 MHz, CD_3_OD) δ 7.40 (s, 1H), 7.34 (s, 1H), 7.25 (d, *J* = 1.9 Hz, 1H), 7.17 (dd, *J* = 8.3, 2.0 Hz, 1H), 7.02 (d, *J* = 8.3 Hz, 1H), 5.20 – 5.11 (m, 1H), 4.63 (dd, *J* = 12.3, 4.4 Hz, 1H), 4.48 (dd, *J* = 12.3, 4.6 Hz, 1H), 4.38 – 4.30 (m, 4H), 3.87 (s, 0.3H), 3.66 (s, 3H), 3.30 – 3.21 (m, 4H), 3.04 – 2.94 (m, 2H), 2.82 – 2.73 (m, 4H), 2.04 – 1.91 (m, 4H), 0.63 (s, 9H).

^13^C NMR (125 MHz, CD_3_OD) δ 170.5, 150.4, 145.6, 144.3, 143.5, 136.2, 132.7, 127.9, 127.4, 123.3, 120.7, 120.4 (q, *^1^J_C-F_* = 318.8 Hz), 119.4, 117.19, 117.16, 116.8, 112.9, 111.9, 104.1, 72.6, 64.6, 64.3, 57.0, 55.0, 51.4, 49.4, 48.9, 34.1, 28.4, 26.9, 21.5, 20.8, 20.7.

HRMS (ESI-TOF) m/z: [M-CF_3_SO_3_]^+^ calcd for: C_34_H_39_N_4_O_5_S; 615.2641 found: 615.2641.

***12-(Tert-butylamino)-6-(2-methoxy-2-oxoethyl)-9-methyl-10-phenyl-2,3,5,6,15,16-hexahydro-1H,14H-pyrido[3,2,1-ij]thiazolo[2'',3'':2',3']imidazo[1',5':4,5][1,4]oxazepino [7,6-f]quinolin-7-ium trifluoromethanesulfonate (6p).***

Yellowish solid (194 mg, 54%). mp 188-190 ^o^C.

IR (neat): 2965, 1733, 1438, 1384, 1251, 1204, 1028 cm^-1^.

^1^H NMR (500 MHz, CD_3_OD) δ 7.65 – 7.58 (m, 5H), 7.30 (s, 1H), 5.17 – 5.10 (m, 1H), 4.63 (dd, *J* = 12.3, 4.3 Hz, 1H), 4.49 (dd, *J* = 12.3, 4.7 Hz, 1H), 3.68 (s, 3H), 3.28 – 3.21 (m, 4H), 3.03 – 2.89 (m, 2H), 2.80 – 2.72 (m, 4H), 2.46 (s, 3H), 2.02 – 1.92 (m, 4H), 0.53 (s, 9H).

^13^C NMR (125 MHz, CD_3_OD) δ 170.5, 150.3, 145.6, 141.5, 132.0, 131.21, 131.19, 129.9, 128.3, 127.7, 127.4, 127.2, 126.3, 120.4 (q, *^1^J_C-F_* = 318.8 Hz), 117.1, 111.9, 104.1, 72.6, 57.0, 54.8, 51.4, 49.4, 48.9, 34.0, 28.4, 26.9, 21.5, 20.8, 20.7, 11.5.

HRMS (ESI-TOF) m/z: [M-CF_3_SO_3_]^+^ calcd for: C_33_H_39_N_4_O_3_S; 571.2742 found: 571.2742.

***12-(Tert-butylamino)-10-(4-fluorophenyl)-6-(2-methoxy-2-oxoethyl)-9-methyl-2,3,5,6,15,16-hexahydro-1H,14H-pyrido[3,2,1-ij]thiazolo[2'',3'':2',3']imidazo [1',5':4,5][1,4]oxazepino[7,6-f]quinolin-7-ium trifluoromethanesulfonate (6q).***

Yellowish solid (202 mg, 55%). mp 188-190 ^o^C.

IR (neat): 2924, 1731, 1505, 1438, 1251, 1222, 1028 cm^-1^.

^1^H NMR (500 MHz, Acetone-d_6_) δ 7.83 – 7.76 (m, 2H), 7.43 – 7.37 (m, 3H), 5.30 – 5.24 (m, 1H), 4.79 (dd, *J* = 12.5, 3.5 Hz, 1H), 4.69 (dd, *J* = 12.5, 5.0 Hz, 1H), 3.69 (s, 0.8H), 3.67 (s, 3H), 3.27 – 3.21 (m, 4H), 3.15 (dd, *J* = 18.0, 3.9 Hz, 1H), 3.04 (dd, *J* = 18.0, 10.0 Hz, 1H), 2.78 – 2.69 (m, 4H), 2.53 (s, 3H), 2.00 – 1.89 (m, 4H), 0.60 (s, 9H).

^13^C NMR (125 MHz, Acetone-d_6_) δ 170.4, 163.5 (d, *^1^J_C-F_* = 248.2 Hz), 150.4, 145.5, 141.9, 133.9 (d, *^2^J_C-F_* = 10.08 Hz), 132.5, 130.3, 128.1, 127.7, 126.8, 123.7 (d, *^3^J_C-F_* = 3.8 Hz), 121.5 (q, *^1^J_C-F_* = 318.8 Hz), 116.6, 115.5, 111.3, 103.1, 72.6, 57.8, 55.0, 54.9, 51.7, 49.5, 48.9, 33.9, 26.9, 21.6, 21.0, 20.9, 12.2.

HRMS (ESI-TOF) m/z: [M-CF_3_SO_3_]^+^ calcd for: C_33_H_38_FN_4_O_3_S; 589.2648 found: 589.2635.

***12-(Tert-butylamino)-10-(2,4-dichlorophenyl)-6-(2-methoxy-2-oxoethyl)-9-methyl-2,3,5,6,15,16-hexahydro-1H,14H-pyrido[3,2,1-ij]thiazolo[2'',3'':2',3']imidazo[1',5':4,5] [1,4]oxazepino[7,6-f]quinolin-7-ium trifluoromethanesulfonate (6r).***

Yellowish solid (228 mg, 58%). mp 209-211 ^o^C.

IR (neat): 2955, 1738, 1580, 1504, 1450, 1277, 1252, 1158, 1028 cm^-1^.

^1^H NMR (500 MHz, CD_3_OD) δ 7.77 (d, *J* = 1.9 Hz, 1H), 7.69 (d, *J* = 5.0 Hz, 1H), 7.59 (dd, *J* = 8.3, 1.9 Hz, 1H), 7.21 (s, 1H), 5.13 – 5.07 (m, 1H), 4.60 (dd, *J* = 11.9, 4.6 Hz, 1H), 4.46 (dd, *J* = 11.9, 6.0 Hz, 1H), 3.69 (s, 3H), 3.62 (s, 0.4 H), 3.29 – 3.21 (m, 4H), 3.07 – 3.00 (m, 2H), 2.82 – 2.73 (m, 4H), 2.41 (s, 3H), 2.03 – .93 (m, 4H), 0.59 (s, 9H).

^13^C NMR (125 MHz, CD_3_OD) δ 171.6, 151.5, 147.2, 142.0, 138.4, 137.6, 136.2, 134.5, 130.7, 130.3, 128.82, 128.78, 128.5, 128.2, 126.6, 121.8 (q, *^1^J_C-F_* = 320.0 Hz), 118.8, 113.9, 106.1, 74.5, 57.7, 55.8, 52.9, 50.8, 50.3, 34.9, 30.1, 28.3, 22.8, 22.1, 22.0, 13.0.

HRMS (ESI-TOF) m/z: [M-CF_3_SO_3_]^+^ calcd for: C_33_H_37_Cl_2_N_4_O_3_S; 639.1963 found: 639.1935.

***10-(4-Bromophenyl)-12-(tert-butylamino)-6-(2-methoxy-2-oxoethyl)-9-methyl-2,3,5,6,15,16-hexahydro-1H,14H-pyrido[3,2,1-ij]thiazolo[2'',3'':2',3']imidazo [1',5':4,5][1,4]oxazepino[7,6-f]quinolin-7-ium trifluoromethanesulfonate (6s).***

Yellowish solid (231 mg, 58%). mp 200-202 ^o^C.

^1^H NMR (500 MHz, CD_3_OD) δ 7.77 (d, *J* = 8.5 Hz, 2H), 7.55 (d, *J* = 8.4 Hz, 2H), 7.26 (s, 1H), 5.17 – 5.09 (m, 1H), 4.63 (dd, *J* = 12.3, 4.5 Hz, 1H), 4.47 (dd, *J* = 12.3, 4.7 Hz, 1H), 3.75 (s, 0.5H), 3.67 (s, 3H), 3.29 – 3.21 (m, 4H), 3.03 – 2.88 (m, 2H), 2.82 – 2.72 (m, 4H), 2.47 (s, 3H), 2.07 – 1.90 (m, 4H), 0.56 (s, 9H).

^13^C NMR (125 MHz, CD_3_OD) δ 170.5, 150.3, 145.7, 141.5, 133.2, 132.6, 131.3, 130.1, 127.6, 127.4, 126.9, 126.2, 123.9, 120.4 (q, *^1^J_C-F_* = 320.0 Hz), 117.2, 112.0, 104.1, 72.7, 56.9, 54.7, 51.4, 49.4, 48.9, 34.1, 28.5, 26.9, 21.5, 20.8, 20.7, 11.6.

HRMS (ESI-TOF) m/z: [M-CF_3_SO_3_]^+^ calcd for: C_33_H_38_BrN_4_O_3_S; 649.1847 found: 649.1848.

***10-(Benzo[d][1,3]dioxol-4-yl)-12-(tert-butylamino)-6-(2-methoxy-2-oxoethyl)-9-methyl-2,3,5,6,15,16-hexahydro-1H,14H-pyrido[3,2,1-ij]thiazolo[2'',3'':2',3']imidazo[1',5':4,5] [1,4]oxazepino[7,6-f]quinolin-7-ium trifluoromethanesulfonate (6t).***

Yellowish solid (194 mg, 51%). mp 174-176 ^o^C.

IR (neat): 2939, 1738, 1487, 1446, 1256, 1201, 1150, 1028 cm^-1^.

^1^H NMR (500 MHz, CD_3_OD) δ 7.30 (s, 1H), 7.13 (d, *J* = 1.5 Hz, 1H), 7.11 – 7.03 (m, 2H), 6.14 – 6.07 (m, 2H), 5.15 – 5.08 (m, 1H), 4.62 (dd, *J* = 12.3, 4.0 Hz, 1H), 4.48 (dd, *J* = 12.3, 4.7 Hz, 1H), 3.67 (s, 3H), 3.28 – 3.20 (m, 4H), 3.03 – 2.86 (m, 2H), 2.80 – 2.73 (m, 4H), 2.46 (s, 3H), 2.03 – 1.92 (m, 4H), 0.62 (s, 9H). ^13^C NMR (125 MHz, CD_3_OD) δ 170.5, 150.3, 149.4, 148.0, 145.6, 141.4, 131.8, 131.0, 127.7, 127.5, 126.0, 125.5, 120.4 (q, *^1^J_C-F_* = 320.0 Hz), 120.3, 117.1, 111.9, 111.4, 107.9, 104.1, 101.9, 72.6, 57.0, 54.8, 51.4, 49.4, 48.9, 34.0, 28.6, 26.9, 21.5, 20.8, 20.7, 11.6.

HRMS (ESI-TOF) m/z: [M-CF_3_SO_3_]^+^ calcd for: C_34_H_39_N_4_O_5_S; 615.2641 found: 615.2659.

***12-(Tert-butylamino)-9-(ethoxycarbonyl)-6-(2-methoxy-2-oxoethyl)-10-phenyl-2,3,5,6,15,16-hexahydro-1H,14H-pyrido[3,2,1-ij]thiazolo[2'',3'':2',3']imidazo [1',5':4,5][1,4]oxazepino[7,6-f]quinolin-7-ium trifluoromethanesulfonate (6u).***

Yellowish solid (210 mg, 54%). mp 182-184 ^o^C.

IR (neat): 3352, 1731, 1613, 1380, 1254, 1149, 1029 cm^-1^.

^1^H NMR (500 MHz, CD_3_OD) δ 7.78 – 7.67 (m, 2H), 7.67 – 7.57 (m, 3H), 7.31 (s, 1H), 5.28 – 5.18 (m, 1H), 4.66 (dd, *J* = 12.5, 4.9 Hz, 1H), 4.52 (dd, *J* = 12.5, 4.2 Hz, 1H), 4.33 – 4.21 (m, 2H), 3.69 (s, 3H), 3.44 (s, 0.6H), 3.29 – 3.21 (m, 4H), 3.02 (dd, *J* = 18.1, 3.8 Hz, 1H), 2.91 (dd, *J* = 18.1, 10.1 Hz, 1H), 2.80 – 2.70 (m, 4H), 2.03 – 1.92 (m, 4H), 1.20 (t, *J* = 7.1 Hz, 3H), 0.55 (s, 9H).

^13^C NMR (125 MHz, CD_3_OD) δ 170.7, 159.4, 150.4, 145.8, 143.4, 139.8, 133.0, 132.4, 130.6, 128.3, 127.8, 127.7, 126.4, 121.1, 120.4 (q, *^1^J_C-F_* = 320.0 Hz), 117.0, 111.6, 103.0, 72.3, 62.5, 57.6, 54.9, 51.5, 49.4, 48.9, 34.0, 28.5, 28.3, 26.8, 21.4, 20.8, 12.7.

HRMS (ESI-TOF) m/z: [M-CF_3_SO_3_]^+^ calcd for: C_35_H_41_N_4_O_5_S; 629.2797 found: 629. 2786.

***12-(Tert-butylamino)-6-(2-methoxy-2-oxoethyl)-9,10-diphenyl-2,3,5,6,15,16-hexahydro-1H,14H-pyrido[3,2,1-ij]thiazolo[2'',3'':2',3']imidazo[1',5':4,5][1,4]oxazepino[7,6-f]quinolin-7-ium trifluoromethanesulfonate (6v).***

Yellowish solid (218 mg, 56%). mp 169-171 ^o^C.

IR (neat): 2945, 1725, 1611, 1505, 1446, 1263, 1149, 1031 cm^-1^.

^1^H NMR (500 MHz, CD_3_OD) δ 7.63 – 7.47 (m, 5H), 7.46 – 7.28 (m, 6H), 5.26 – 5.17 (m, 1H), 4.68 (dd, *J* = 12.3, 4.1 Hz, 1H), 4.53 (dd, *J* = 12.3, 4.4 Hz, 1H), 3.67 (s, 3H), 3.60 (s, 0.3H), 3.30 – 3.20 (m, 4H), 3.07 – 2.91 (m, 2H), 2.83 – 2.71 (m, 4H), 2.05 – 1.93 (m, 4H), 0.55 (s, 9H).

^13^C NMR (125 MHz, CD_3_OD) δ 170.6, 150.4, 145.7, 142.0, 132.2, 131.8, 130.8, 130.0, 129.9 (2C), 129.6, 129.0, 128.8, 128.4, 128.0, 127.6, 127.3, 120.4 (q, *^1^J_C-F_* = 320.0 Hz), 117.1, 111.8, 103.8, 72.5, 57.3, 54.8, 51.4, 49.4, 48.9, 34.0, 28.5, 26.9, 21.5, 20.8, 20.7.

HRMS (ESI-TOF) m/z: [M-CF_3_SO_3_]^+^ calcd for: C_38_H_41_N_4_O_3_S; 633.2899 found: 633.2848.

***12-(Tert-butylamino)-6-(2-methoxy-2-oxoethyl)-9,10-bis(4-methoxyphenyl)-2,3,5,6,15,16-hexahydro-1H,14H-pyrido[3,2,1-ij]thiazolo[2'',3'':2',3']imidazo[1',5':4,5][1,4]oxazepino [7,6-f]quinolin-7-ium trifluoromethanesulfonate (6w).***

Yellowish solid (227 mg, 54%). mp 186-188 ^o^C.

IR (neat): 3344, 1727, 1608, 1498, 1451, 1266, 1248, 1140, 1032 cm^-1^.

^1^H NMR (500 MHz, CD_3_OD) δ 7.50 – 7.41 (m, 2H), 7.34 (s, 1H), 7.24 (d, *J* = 8.2 Hz, 2H), 7.06 (d, *J* = 7.6 Hz, 2H), 6.93 (d, *J* = 8.2 Hz, 2H), 5.23 – 5.13 (m, 1H), 4.67 (d, 1H), 4.52 (dd, *J* = 12.1, 3.8 Hz, 1H), 3.87 (s, 3H), 3.81 (s, 3H), 3.66 (s, 3H), 3.20 – 3.21(m, 4H), 3.07 – 2.97 (m, 2H), 2.84 – 2.72 (m, 4H), 2.05 – 1.93 (m, 4H), 0.58 (s, 9H).

^13^C NMR (125 MHz, CD_3_OD) δ 172.0, 162.7, 162.3, 151.8, 147.0, 143.0, 134.5, 133.2, 132.3, 131.5, 130.4, 129.4, 128.9, 122.4, 120.6, 120.4 (q, *^1^J_C-F_* = 320.0 Hz), 118.5, 115.6, 115.2, 113.2, 105.4, 74.0, 58.5, 56.4, 56.0, 55.9, 52.8, 50.8, 50.3, 35.5, 30.0, 28.3, 22.9, 22.2, 22.1.

HRMS (ESI-TOF) m/z: [M-CF_3_SO_3_]^+^ calcd for: C_40_H_45_N_4_O_5_S; 693.3110 found: 693.3083.

***14-bromo-18-(tert-butylamino)-10-(2-methoxy-2-oxoethyl)-2,3,6,7,9,10-hexahydro-1H,5H-benzo[4'',5'']thiazolo[2'',3'':2',3']imidazo[1',5':4,5][1,4]oxazepino[7,6-f]pyrido[3,2,1-ij]quinolin-11-ium trifluoromethanesulfonate (6x).***

Yellowish solid (185 mg, 49%). mp 153-155 ^o^C.

IR (neat): 3339, 1733, 1613, 1518, 1461, 1254, 1168, 1028 cm^-1^.

^1^H NMR (500 MHz, CD_3_OD) δ 8.65 (d, *J* = 8.8 Hz, 1H), 8.40 (d, *J* = 1.8 Hz, 1H), 7.92 (dd, *J* = 8.8, 1.9 Hz, 1H), 7.30 (s, 1H), 5.24 – 5.14 (m, 1H), 4.71 (dd, *J* = 12.1, 4.7 Hz, 1H), 4.47 (dd, *J* = 12.1, 4.5 Hz, 1H), 3.63 (s, 3H), 3.31 – 3.21 (m, 4H), 3.03 – 2.87 (m, 2H), 2.87 – 2.73 (m, 4H), 2.07 – 1.93 (m, 4H), 1.11 (s, 9H). ^13^C NMR (125 MHz, CD_3_OD) δ 170.3, 150.3, 145.9, 142.8, 133.3, 131.6, 131.0, 130.6, 128.7, 127.3, 127.0, 120.4 (q, *^1^J_C-F_* = 318.8 Hz), 119.9, 118.0, 117.5, 112.4, 104.3, 72.8, 57.1, 56.0, 51.4, 49.4, 48.9, 34.2, 28.9, 26.9, 21.4, 20.7, 20.6.

HRMS (ESI-TOF) m/z: [M-CF_3_SO_3_]^+^ calcd for: C_30_H_34_BrN_4_O_3_S; 609.1534 found: 609.1545.

***18-(Tert-butylamino)-10-(2-methoxy-2-oxoethyl)-14-(methylsulfonyl)-2,3,6,7,9,10-hexahydro-1H,5H-benzo[4'',5'']thiazolo[2'',3'':2',3']imidazo[1',5':4,5][1,4]oxazepino[7,6-f]pyrido[3,2,1-ij]quinolin-11-ium trifluoromethanesulfonate (6y).***

Yellowish solid (94 mg, 25%). mp 170-172 ^o^C.

IR (neat): 3359, 1741, 1618, 1524, 1372, 1253, 1151, 1029 cm^-1^.

^1^H NMR (500 MHz, CD_3_OD) δ 8.97 (d, *J* = 8.7 Hz, 1H), 8.85 (s, 1H), 8.37 – 8.30 (m, 1H), 7.32 (s, 1H), 5.24 (dd, *J* = 9.2, 4.5 Hz, 1H), 4.73 (dd, *J* = 12.0, 4.5 Hz, 1H), 4.50 (dd, *J* = 12.0, 4.3 Hz, 1H), 3.63 (s, 3H), 3.27 (m, 7H), 3.04 –2.94 (m, 2H), 2.81 (dd, *J* = 12.8, 6.3 Hz, 4H), 2.06 – 1.96 (m, 4H), 1.13 (s, 9H).

^13^C NMR (125 MHz, CD_3_OD) δ 171.7, 151.7, 147.4, 145.8, 141.1, 137.0, 135.2, 131.9, 130.3, 128.4, 127.9, 126.2, 121.8 (q, *^1^J_C-F_* = 318.8 Hz), 118.9 (2C), 113.8, 105.5, 74.1, 58.7, 57.5, 52.8, 50.8, 50.3, 44.4, 35.6, 30.3, 28.3, 22.8, 22.1 (2C).

HRMS (ESI-TOF) m/z: [M-CF_3_SO_3_]^+^ calcd for: C_31_H_37_N_4_O_5_S_2_; 609.2205 found: 609.2185.

**General Reaction Procedure for the Preparation of Compounds 7a-c.** Compounds **7a-c** were prepared according to a precedent procedure and the spectroscopic data for the known compounds (**7a** and **7b**) are matching that published in reference 5a.

***Methyl (E)-4-(2-formyl-1H-pyrrol-1-yl)but-2-enoate (7a).***

Yellowish solid (213 mg, 55% yield). mp 55-57 ^o^C

IR (neat): 2952, 2923, 1714, 1659, 1435, 1351, 1244, 1160, 1079, 748 cm^-1^.

^1^H NMR (500 MHz, CDCl_3_) δ 9.56 (s, 1H), 7.05 (dt, *J* = 15.6, 4.8 Hz, 1H), 7.00 (dd, *J* = 4.0, 1.7 Hz, 1H), 6.96 – 6.92 (m, 1H), 6.32 (dd, *J* = 4.0, 2.5 Hz, 1H), 5.55 (dt, *J* = 15.6, 1.9 Hz, 1H), 5.16 (dd, *J* = 4.8, 1.9 Hz, 2H), 3.72 (s, 3H).

^13^C NMR (125 MHz, CDCl_3_) δ 179.5, 166.3, 143.8, 131.3, 125.0, 121.9, 110.6, 51.8, 49.3

HRMS (ESI-TOF) m/z: [M+Na]^+^ calcd for: C_10_H_11_NO_3_Na; 216.0636 found: 216.0613.

***Methyl (E)-4-(2-formyl-1H-indol-1-yl)but-2-en*** ***(7b).***

Greenish solid (218 mg, 45% yield). mp 100-102 ^o^C

IR (neat): 2919, 1719, 1660, 1429, 1255, 1163, 1127, 852, 820, 738 cm^-1^.

^1^H NMR (500 MHz, CDCl_3_) δ 9.89 (s, 1H), 7.79 (d, *J* = 8.1 Hz, 1H), 7.47 – 7.42 (m, 1H), 7.38 – 7.28 (m, 2H), 7.26 – 7.21 (m, 1H), 7.14 – 7.07 (dt, *J* = 15.6, 4.6 Hz, 1H), 5.48 (td, *J* = 15.6, 1.9 Hz, 1H), 5.41 (dd, *J* = 4.6, 1.9 Hz, 2H), 3.69 (s, 3H).

^13^C NMR (125 MHz, CDCl_3_) δ 182.6, 166.3, 143.3, 140.2, 135.0, 127.6, 126.6, 123.8, 121.7, 121.6, 118.6, 110.5, 51.7, 45.3.

HRMS (ESI-TOF) m/z: [M+Na]^+^ calcd for: C_14_H_13_NO_3_Na; 266.0793 found: 266.0767.***Methyl (E)-4-(2-Formyl-1H-imidazol-1-yl)but-2-enoate (7c).***

Yellowish solid (155 mg, 40% yield). mp 60-62 ^o^C.

IR (neat): 3113, 2953, 2847, 1717, 1679, 1410, 1277, 1197, 1025, 766 cm^-1^.

^1^H NMR (400 MHz, CDCl_3_) δ 9.82 (s, 1H), 7.35 (d, *J* = 0.8 Hz, 1H), 7.16 (s, 1H), 7.05 – 6.97 (m, 1H), 5.70 – 5.63 (m, 1H), 5.22 (dd, *J* = 5.2, 1.8 Hz, 2H), 3.74 (s, 3H).

^13^C NMR (101 MHz, CDCl_3_) δ 182.0, 165.8, 143.1, 141.4, 132.1, 126.1, 123.1, 51.9, 47.9.

HRMS (ESI-TOF) m/z: [M+H]^+^ calcd for: C_9_H_11_N_2_O_3_; 195.0769 found: 195.0748.

**General Reaction Procedure for the Preparation of Compounds 8a-c.** Ytterbium triflate (20 mol%) and sodium sulfate (1.0 mmol) were added to a solution of aldehyde (**7**, 0.5 mmol), 2-aminoazine (**5c,** 0.5 mmol) in MeOH:DCM (1.5:0.5 mL) at rt. After 45 mins, tert-Butyl isocyanide (0.55 mmol) was introduced and stirring was continued for 12h. After completion of step-1, an additional 30 mol% of ytterbium triflate was added and the reaction was continued at 70 °C for 6h. After completion, MeOH and DCM were removed and the crude was purified on flash column chromatography, using a gradient of EtOAC/hexane or MeOH/DCM as eluents to produce compounds **8a-c**.

***12-(Tert-butylamino)-6-(2-methoxy-2-oxoethyl)-9-methyl-10-phenyl-5,6-dihydropyrrolo [1,2-a]thiazolo[2',3':2,3]imidazo[5,1-c]pyrazin-7-ium trifluoromethanesulfonate (8a).***

Yellowish solid (167 mg, 55%). mp 140-142 ^o^C.

IR (neat): 3337, 1731, 1635, 1521, 1439, 1255, 1149, 1028 cm^-1^.

^1^H NMR (500 MHz, CD_3_OD) δ 7.64 – 7.57 (m, 5H), 7.02 – 6.98 (m, 1H), 6.86 (d, *J* = 2.9 Hz, 1H), 6.31 (t, *J* = 2.9 Hz, 1H), 5.41 – 5.35 (m, 1H), 4.60 – 4.48 (m, 2H), 3.92 (s, 0.7H), 3.71 (s, 3H), 2.92 (dd, *J* = 17.4, 4.5 Hz, 1H), 2.70 (dd, *J* = 17.4, 8.5 Hz, 1H), 2.48 (s, 3H), 0.70 (s, 9H).

^13^C NMR (125 MHz, CD_3_OD) δ 170.6, 141.8, 131.4, 131.3, 129.8, 128.1, 127.0, 126.7, 125.3, 124.3, 122.9, 120.4 (q, *^1^J_C-F_* = 320.0 Hz), 117.0, 109.4, 109.2, 56.0, 52.8, 51.5, 48.1, 35.1, 28.8, 11.8.

HRMS (ESI-TOF) m/z: [M-CF_3_SO_3_]^+^ calcd for: C_25_H_29_N_4_O_2_S; 449.2011 found: 449.2017.

***13-(Tert-butylamino)-5-(2-methoxy-2-oxoethyl)-2-methyl-1-phenyl-5,6-dihydrothiazolo [2'',3'':2',3']imidazo[5',1':3,4]pyrazino[1,2-a]indol-4-ium trifluoromethanesulfonate (8b).***

White solid (145 mg, 45%). mp 196-198 ^o^C.

IR (neat): 3337, 1740, 1490, 1437, 1262, 1203, 1150, 1031 cm^-1^.

^1^H NMR (500 MHz, CD_3_OD) δ 7.70 – 7.60 (m, 6H), 7.53 (d, *J* = 8.2 Hz, 1H), 7.32 (t, *J* = 7.2 Hz, 1H), 7.27 (s, 1H), 7.18 (t, *J* = 7.2 Hz, 1H), 5.58 – 5.52 (m, 1H), 4.95 (dd, *J* = 13.5, 1.4 Hz, 1H), 4.53 (dd, *J* = 13.5, 3.8 Hz, 1H), 3.65 (s, 3H), 2.97 (dd, *J* = 17.3, 4.4 Hz, 1H), 2.77 (dd, *J* = 17.3, 8.4 Hz, 1H), 2.51 (s, 3H), 0.75 (s, 9H).

^13^C NMR (125 MHz, CD_3_OD) δ 170.6, 142.7, 136.6, 131.4, 131.3, 129.9 (2C), 128.2 (2C), 128.0, 127.3, 126.9, 123.5, 123.4, 122.6, 121.1, 120.7, 120.4 (q, *^1^J_C-F_* = 320.0 Hz), 109.0, 102.1, 56.6, 53.1, 51.4, 35.4, 28.8, 11.9.

HRMS (ESI-TOF) m/z: [M-CF_3_SO_3_]^+^ calcd for: C_29_H_31_N_4_O_2_S; 499.2167 found: 499.2147.

***12-(Tert-butylamino)-6-(2-methoxy-2-oxoethyl)-9-methyl-10-phenyl-5,6-dihydroimidazo [1,2-a]thiazolo[2',3':2,3]imidazo[5,1-c]pyrazin-7-ium trifluoromethanesulfonate (8c).***

White solid (152 mg, 51%). mp 180-182 ^o^C.

IR (neat): 3374, 1727, 1439, 1365, 1242, 1149, 1029 cm^-1^.

^1^H NMR (500 MHz, CD_3_OD) δ 7.66 – 7.57 (m, 5H), 7.45 – 7.41 (m, 1H), 7.30 – 7.25 (m, 1H), 5.57 – 5.48 (m, 1H), 4.78 – 4.65 (m, 2H), 3.72 (s, 3H), 2.97 (dd, *J* = 17.5, 3.6 Hz, 1H), 2.77 (dd, *J* = 17.5, 8.3 Hz, 1H), 2.50 (s, 3H), 0.70 (s, 9H).

^13^C NMR (125 MHz, CD_3_OD) δ 170.4, 143.6, 134.0, 131.6, 131.5, 129.8, 129.2, 129.1, 127.8, 127.7, 126.5, 121.0, 120.6 (q, *^1^J_C-F_* = 320.0 Hz), 119.9, 56.3, 52.5, 51.6, 46.9, 35.2, 28.1, 12.0.

HRMS (ESI-TOF) m/z: [M-CF_3_SO_3_]^+^ calcd for: C_24_H_28_N_5_O_2_S; 450.1963 found: 450.1976.
